# Biologically active secondary metabolites from white-rot fungi

**DOI:** 10.3389/fchem.2024.1363354

**Published:** 2024-03-13

**Authors:** Orkun Pinar, Susana Rodríguez-Couto

**Affiliations:** Department of Separation Science, LUT School of Engineering Science, Lappeenranta-Lahti University of Technology LUT, Mikkeli, Finland

**Keywords:** white-rot fungi, secondary metabolites, biologically active compounds, bioactive properties, therapeutic substances

## Abstract

In recent years, there has been a considerable rise in the production of novel metabolites derived from fungi compared to the ones originating from bacteria. These organic substances are utilized in various sectors such as farming, healthcare, and pharmaceutical. Since all dividing living cells contain primary metabolites, secondary metabolites are synthesized by utilizing intermediate compounds or by-products generated from the primary metabolic pathways. Secondary metabolites are not critical for the growth and development of an organism; however, they exhibit a variety of distinct biological characteristics. White-rot fungi are the only microorganisms able to decompose all wood components. Hence, they play an important role in both the carbon and nitrogen cycles by decomposing non-living organic substrates. They are ubiquitous in nature, particularly in hardwood (e.g., birch and aspen) forests. White-rot fungi, besides ligninolytic enzymes, produce different bioactive substances during their secondary metabolism including some compounds with antimicrobial and anticancer properties. Such properties could be of potential interest for the pharmaceutical industries. Considering the importance of the untapped biologically active secondary metabolites from white-rot fungi, the present paper reviews the secondary metabolites produced by white-rot fungi with different interesting bioactivities.

## 1 Introduction

Biologically active compounds are synthesized mostly by fungi, bacteria, archaea, and plants. These compounds possess different properties that make them suitable for various applications including drug development with anti-glycaemic, anticancer, antibiotic, antiviral, anti-inflammatory, enzyme inhibiting, hypercholesteremic, immunomodulator, immunosuppressant, cardiovascular, antithrombotic, antidiabetic, antihypertensive, neuropathic, and anti-infective characteristics for humans (Basit et al., 2021; Conrado et al., 2022; Kijpornyongpan et al., 2022). Starting from the 1940s, microorganisms have played a significant role in uncovering important sources of various natural products used in the agrochemical, cosmetic, pharmaceutical, and food industries (Baltz, 2019; Gakuubi et al., 2022). Because of their exceptional biological activities, these compounds have gained the attention of researchers in various fields, including those investigating natural product-derived medicines as well as chemists, biochemists, and microbiologists. In addition, recently, metabolic engineers seek to enlighten regulations, pathways, and gene clusters to produce bioactive compounds efficiently using suitable host organisms with the assistance of genome sequencing to bioinformatics, transcriptomics, metabolomics, and proteomics (Tsukada et al., 2020; Kijpornyongpan et al., 2022). In this sense, microbial secondary metabolites are beneficial for human wellbeing, owing to their extensive usage in various biological processes spanning across agriculture, medical sciences, food technology, and chemical industry (Yadav, 2021). More specifically, among the different existing microorganisms, fungi have emerged as promising candidates for the discovery of novel biologically active compounds because of their varied pharmacological activities.

The advantageous effects of fungi on human health have been mainly associated with the abundance of various bioactive compounds, including carbohydrates, proteins, amino acids, unsaturated fatty acids, vitamins, and minerals (Gebreyohannes and Sbhatu, 2023) together with bioactive secondary metabolites. The therapeutic use of fungal species can be traced back to as early as 3000 BC. Thus, macrofungi such as *Ganoderma lucidum* (*G. lucidum*), *Lentinus edodes* (*L. edodes*), *Fomes fomentarius*, and *Fomitopsis officinalis* were used as remedies for different diseases in some East countries (Wasser, 2002). Medical professionals have been aware for over five thousand years regarding the presence of immune-enhancing and defensive attributes in fungal species (Ribka et al., 2021). In the literature, reports indicate that a significant number of approved medications are sourced from nature, with approximately 25% of the one million natural compounds examined showing biological activities. Among these compounds, around 60% originated from plants, while the remaining is derived from microbes. Notably, fungi contribute to approximately 42% of microbial resources, underscoring their significance in the exploration and identification of novel molecules (Bharatiya et al., 2021). Furthermore, until 2019, approximately 35% of the naturally derived products approved by the US Food and Drug Administration (FDA) contributed to the development of pharmaceuticals (Shankar and Sharma, 2022). Moreover, fungi are also able to synthesize various biologically active compounds such as pigments, dyes, antioxidants, nutraceuticals, dietary supplements, polysaccharides, and industrial enzymes (Al-Mousa et al., 2022a; Al-Mousa et al., 2022b; Hassane et al., 2022; Mohamed et al., 2022). These fungal products are not only crucial for functional food and nutrition but also serve as important sources of pharmacological/medicinal substances (Keller, et al., 2005; Hoeksma et al., 2019; Fukushima-Sakuno, 2020; Elhusseiny et al., 2021; Bhambri et al., 2022; Krupodorova et al., 2022). These intriguing scientific discoveries have garnered significant interest from researchers who are exploring the potential applications of these metabolites.

Metabolites are small and intermediate metabolism products that serve various purposes in organisms. These products are classified into primary and secondary metabolites. In this sense, primary metabolites are produced for growth, development, and survival and consist of amino acids, sugars, vitamins, lipids, nucleotides, and carbohydrates, which have critical duties in different metabolic processes including respiration and nutrient consumption. On the other hand, secondary metabolites are not integral components of the metabolic pathways; instead, they are synthesized as byproducts in terms of defense mechanisms and derived from primary metabolism. These compounds exhibit diverse biological functions and result from metabolic reactions that are non-essential for growth and reproduction of organisms. The production of secondary metabolites provides a competitive advantage to the organism by increasing the tolerance to environmental stresses and extreme conditions, and thereby indirectly influencing ecological dynamics (Devi and Krishnakumari, 2015; Daley et al., 2017; Thirumurugan et al., 2018; Conrado et al., 2022; Rodríguez-Berríos et al., 2023; Sharma et al., 2023). “Secondary metabolites” term was first introduced by the Nobel Prize laureate Albrecht Kossel in 1891 and the botanist Friedrich Czapek further created the term “secondary modifications” in his work related to plant nitrogen metabolism in the 1920s (Henriksen et al., 2022). Secondary metabolites are a diverse group of organic compounds primarily derived from various sources such as plants, fungi, and bacteria. These bioactive molecules are generally low molecular weight compounds (Molecular weight <1,500 Da) (Zerva et al., 2020a). They present scientific interest due to their multiple applications in industries (e.g., textile, functional food innovation, flavoring, glues, oil). Additionally, they hold promising potential for the development of novel pharmaceuticals, antibiotics, insecticides for pest control, and herbicides targeting unwanted plant growth (Devi and Krishnakumari, 2015; Devi et al., 2022; Shankar and Sharma, 2022).

The biosynthesis of fungal secondary bioactive metabolites is typically based on the mevalonic acid pathway, the acetate pathway, and carbohydrate/polysaccharide synthesis (Kundu, 2021). These fungal-derived bioactive compounds can be divided into high and low molecular weight. The former predominantly comprises polysaccharides and enzymes, while the latter encompasses terpenoids, phenols, and indoles, among others (Ziaja-Sołtys et al., 2022). Conrado et al. (2022) reported that out of the 500,000 secondary metabolites, approximately 70,000 are sourced from microorganisms. Among these compounds, around 33,500 exhibit bioactive properties, and about 47% of these bioactive compounds originate from fungal strains. Up to date, numerous fungal species, particularly filamentous fungi found within the basidiomycetes class, can be considered for an extensive range production of secondary metabolites with significant biological activities (Teoh et al., 2011; Patel and Goyal, 2012). Basidiomycetes are known for their ability to produce numerous secondary metabolites that exhibit antioxidant (Jayakumar et al., 2009), antimicrobial (Bala et al., 2011), anti-inflammatory (Liu et al., 2015), antifungal (Sidorova and Voronina, 2019), and antiviral (Krupodorova et al., 2014) properties. Moreover, they can also produce cytotoxic compounds with the potential use as anticancer agents and immunomodulating polysaccharides. Additionally, some of these metabolites have hallucinogenic effects while others serve as sources for plant growth regulators or flavors (Prasher and Manju, 2019; Halabura et al., 2023).

In recent years, microorganisms belonging to basidiomycetes have become very promising for red (medical) biotechnology (Mizerska-Dudka et al., 2015) and cosmeceuticals (Zerva et al., 2020b). In this sense, white rot fungi are a prominent group within the phylum basidiomycota, which are saprotrophic organisms in the fungal kingdom, encompassing approximately 30%–32% of fungal diversity with an estimated 30,000 distinct species. These fungi can degrade all components of plant cell wall through various mechanisms including extracellular enzymatic processes as well as non-enzymatic ones such as reactive oxygen species (Kijpornyongpan et al., 2022). Due to the distinct characteristics of white rot fungi, these microorganisms and their extracellular enzymes that primarily include lignin peroxidases (LiPs, EC 1.11.1.14), manganese-dependent peroxidases (MnPs, EC 1.11.1.13), and laccases (benzenediol: oxygen oxidoreductases, EC 1.10.3) along with additional enzymes such as peroxidase-generating oxidases and mycelium-associated dehydrogenases (Martínez et al., 2005) have been recognized for their potential in various biotechnological applications. With the aid of these enzymes, white-rot fungi possess the capacity to break down intricate plant cell wall polymers such as cellulose, hemicellulose, and lignin. White-rot fungi represent the only known group of organisms that have evolved to effectively break down lignin into carbon dioxide (CO_2_) and water (H_2_O) which contributes significantly to Earth’s ecosystem (Floudas et al., 2012; Kundu, 2021; Llanos-López et al., 2023).

White rot fungi possess a significant capacity to produce numerous enzymes and secondary metabolites, exhibiting potential in the fields of nutrition, medicine, and degradation. These secondary metabolites, including terpenoids, polyphenols, sterols, flavonoids, alkaloids, derivatives of benzoic acid, quinolones, anthraquinones, and lactones possess bioactive characteristics (Jaszek et al., 2013; Jaszek et al., 2014; Fernando et al., 2016; Bogale, 2020; Kundu and Khan, 2021; Mahuri et al., 2023). Moreover, considering the economic significance and the intention of applications based on biologically active secondary metabolites, there has been a rising global interest in these compounds produced by white rot fungi. Thus, recognizing the great potential of biologically active secondary metabolites from white-rot fungi, this review focuses on exploring the secondary metabolites generated by them, emphasizing their fascinating bioactive properties.

## 2 Biologically active secondary metabolites produced by white-rot fungi

**FIGURE 1:**
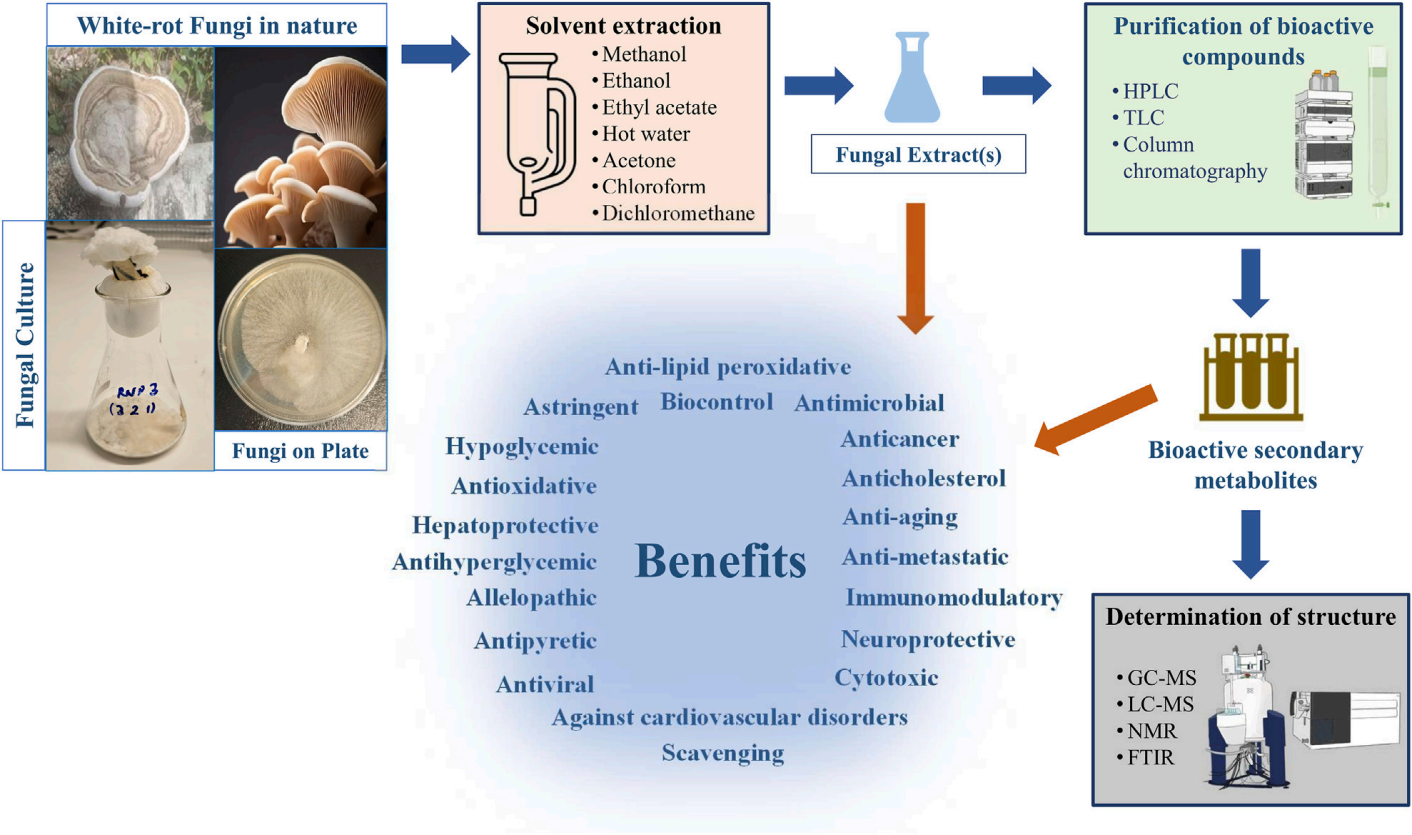
General processing steps of biologically active secondary metabolites production from white-rot fungi.

Secondary bioactive metabolites produced by white rot fungi have significant potential of applicability in various sectors such as pharmaceutical production (Zheng et al., 2010), biobleaching processes in pulp and paper industries (Jerusik, 2010), wastewater treatment approaches (Muszyńska et al., 2019), enhancing digestibility of cellulose and lignin in animals (Yilkal, 2015), generation of renewable resources from lignocellulosic materials, and bioremediation technologies (Korcan et al., 2012; Contreras et al., 2023) (Figure 1). In the literature, several works have revealed the production of biologically active secondary metabolites of white-rot fungi (Table 1). Moreover, the chemical structures of some bioactive secondary metabolites from white-rot fungi are illustrated in Table 2. Considering this, the *Schizophyllum* genus has been an important white-rot fungal genus with the capability of producing bioactive secondary metabolites. The combined treatment of radiotherapy and sizofiran, a polysaccharide extract from the culture broth of *Schizophyllum commune* (*S. commune*), resulted in a significantly higher 5-year survival rate with 90 patients compared to 82 patients treated with radiotherapy alone. Sizofiran demonstrated promising potential as an immunotherapeutic agent in the treatment of cervical carcinoma (Miyazaki et al., 1995).

**TABLE 1:** Bioactive secondary metabolites from white-rot fungi and their benefits.

Strain name	Bioactive compound(s)	Benefits	References
*S. commune*	Sizofiran	Anticancer	Miyazaki et al. (1995)
*S. commune*	Schizostatin	Anticholesterol	Tanimoto et al. (1996)
*S. commune*	Phenyl benzoate (C_13_H_10_O_2_), 4-(phenyl methoxy) phenol (C_13_H_12_O_2_), pyrrolo (1, 2-a) piperazine-3, 6-dione (C_7_H_10_O_2_N_2_), gallic acid, and L-ascorbic acid	Antioxidative	Tripathi and Tiwary (2013)
		Antibacterial	
*S. commune*	Gallic acid, catechin, chlorogenic acid, epicatechin, caffeic acid, coumaric acid, rutin quercetin, and kaempferol	Antioxidative Biocontrol agent	Kaur et al. (2018)
*S. commune*	Phenolics, flavonoids, alkaloids, tannins, and saponins	Antioxidative	Kumar et al. (2018)
*S. commune*	Schizostatin	Antimicrobial	Dutta et al. (2019)
*P. ostreatus*	Lovastatin	Anticholesterol	Alam et al. (2009)
*P. sajor-caju*			
*P. florida*			
*P. florida*	Not determined	Antibacterial	Fagade and Oyelade (2009)
*P. fulvus*			
*P. pulmonarius*	Total phenols and flavonoids	Antioxidative Antimicrobial	Ramesh and Pattar (2010)
*P. ostreatus*	Not determined	Antihyperglycemic	Ghaly et al. (2011)
*P. ostreatus*	Polysaccharides	Antioxidative	Zhang et al. (2012)
*P. sajor-caju*	Exopolysaccharide (EPS) and intracellular polysaccharides (IPSs)	Anticancer	Assis et al. (2013)
*P. ostreatus*	β-glucan	Antioxidative	Mitra et al. (2013)
*P. cystidiosus*	Alkaloids, flavonoids, saponins, and terpenoids	Antimicrobial	Kalaw and Albinto (2014)
		Antioxidative	
*P. ostreatoroseus*	Free sugars, organic acids, phenolic compounds, and tocopherols	Antioxidative	Corrêa et al. (2015)
		Anti-inflammatory Antimicrobial	
*P. sajor caju*	Phenols, flavonoids, tannins, and alkaloids	Antimicrobial	Devi and Krishnakumari (2015)
		Anticancer	
		Antipyretic	
		Astringent	
		Antiviral	
*P. ostreatus*	Phenols and flavonoids	Antioxidative Antimicrobial	Chowdhury et al. (2015)
*P. ostreatus*	Phenolic acids, resveratrol, triterpenic compounds, and ergosterol	Antioxidative	Koutrotsios et al. (2018)
*P. eryngii*			
*P. nebrodensis*			
*P. ostreatus*	Flavonoids, total phenols	Antioxidative	Beltrán Delgado et al. (2021)
*P. pulmonarius*	Exopolysaccharides	Antioxidative	Ogidi et al. (2020)
		Antimicrobial	
*P. ostreatus*	Phenolics, and flavonoids including catechin, kaempferol, apigenin	Anticancer, antiviral, antioxidative	Elhusseiny et al. (2021)
*C. indica*	Phenol, glycerine, pimelic ketone, D-ribonic acid, methyl myristate, palmitic acid methyl ester, oleic acid ethyl ester, lauramide, oleic acid amide, 1,2-cyclododecanediol, resorcinol, and phytol	Antimicrobial	Ogidi et al. (2020)
		Antioxidative	
		Anti-inflammatory	
		Immunomodulator	
		Antitumor	
*L. edodes*	Lentinan	Anticancer	Oba et al. (2009)
*L. edodes*	High phenols and flavonoids	Antioxidative	Chowdhury et al. (2015)
		Antimicrobial	
*L. edodes*	Ergosterol and trilinolein	Antioxidative	Resurreccion et al. (2016)
		Anticancer	
		Against cardiovascular disorders	
*L. tigrinus*	Not determined	Antioxidative	Sevindik (2018a)
		Antimicrobial	
*L. edodes*	Catechin and quercetin	Anticancer	Elhusseiny et al. (2021)
		Antiviral	
		Antioxidative	
*L. swartzii*	Not determined	Antioxidative	Austria et al. (2021)
		Antidiabetic	
*L. squarrosulus*	Not determined	Antioxidative	Muslihin et al. (2022)
*C. versicolor*	Extracellular polysaccharopeptides (PSP)	Immunomodulator	Lin et al. (2008)
*P. strigellus*	Panapophenanthrin, panepophenanthrin, and dihydrohypnophilin	Antimicrobial, anticancer	Llanos-López et al. (2023)
*H. tessulatus*	Phenols and flavonoids	Antioxidative	Chowdhury et al. (2015)
		Antimicrobial	
*C. cylindracea*	Not determined	Antioxidative	Sevindik et al. (2018)
		Anti-inflammatory	
		Anticancer	
*G. frondosa*	Not determined	Anticancer	Masuda et al. (2008)
		Anti-metastatic	
*G. frondosa*	Not determined	Anticancer	Masuda et al. (2009)
		Anti-metastatic	
*G. frondosa*	Not determined	Anticancer	Deng et al. (2009)
*G. frondosa*	Water-soluble polysaccharides	Anti-inflammatory	Su et al. (2020)
		Anticancer	
*O. olearius*	Irofulven	Anticancer	Eckhardt et al. (2000)
*P. chrysosporium P.brevispora P.floridensis*	Not determined	Antioxidative	Chandra et al. (2019)
*H. erinaceus*	Erinacine A	Neuroprotective	Lee et al. (2014)
*H. erinaceus*	Hericenones C, D, and F	Anti-inflammatory	Lee et al. (2016)
*H. erinaceus*	Erinacine A	Neuroprotective	Chang et al. (2016)
*H. erinaceus*	Terpenoid backbones, diterpenoids, sesquiterpenes, and polyketides	Neuroprotective	Chen et al. (2017)
*H. erinaceus*	4-chloro-3,5-dimethoxybenzoic methyl ester, 3-(hydroxymethyl)-2-furaldehyde, erinacine A, erinacerin G, herierin III, and herierin IV	Neuroprotective Neuritogenic	Zhang et al. (2017)
*H. erinaceus*	Erinacine A and S	Anti-neurodegenerative Neuroprotective	Tzeng et al. (2018)
*H. erinaceus*	Erinacine A, hericenone C, and hericenones D	Anti-aging	Ratto et al. (2019)
*H. erinaceus*	Erinacine A, hericenone C, hericenone D, and ergothioneine	Anti-aging, Neuroprotective	Roda et al. (2021)
*H. erinaceus*	Ergothioneine	Anti-aging, Neuroprotective	Roda et al. (2022)
*H. erinaceus*	Ergothioneine	Anti-aging, Neuroprotective	Roda et al. (2023)
*I. lacteus*	Irpexlacte A-D, irlactin E, and 3β-hydroxycinnamolide	Antioxidative	Duan et al. (2019)
		Antimicrobial	
*P. pini*	Gallic acid, catechin, chlorogenic acid, epicatechin, caffeic acid, umbelliferone, coumaric acid, tert-butyl-hydroquinone, and quercetin	Antioxidative	Devi et al. (2022)
*B. adusta*	Not determined	Antioxidative	Ildız et al. (2022)
		Antimicrobial	
*H. myxotricha*	Not determined	Antioxidative	Krupodorova et al. (2022)
		Antimicrobial	
*C. unicolor*	Crude endopolysaccharides (c-EPL) and low molecular weight compound (ex-LMS)	Antioxidative	Jaszek et al. (2013)
		Antibacterial	
*C. unicolor*	Endopolysaccharides (c-EPL) and a low molecular weight metabolites (ex-LMS)	Antiviral	Mizerska-Dudka et al. (2015)
		Anticancer Immunostimulatory Antiproliferative	
*C. unicolor*	Not determined	Antioxidative	Sevindik (2018b)
		Antimicrobial	
*C. unicolor*	Low molecular weight compounds	Anticancer	Matuszewska et al. (2019)
		Antioxidative	
*A. fuscosuccinea*	Alkaloids and tannins glycosides	Antimicrobial	Romorosa et al. (2017)
*C. comatus*	Alkaloids, flavonoids, saponins, and terpenoids, steroids and cardiac glycosides	Antimicrobial	Kalaw and Albinto (2014)
		Antioxidative	
*C. comatus*	4-hydroxybenzoic acid, protocatechuic acid, cinnamic acid, p-coumaric acid, caffeic acid, and quinic acid	Antioxidative	Stilinović et al. (2020)
		Hepatoprotective	
*P. ribis*	Carbohydrates, proteins, amino acids, lipids, alkaloids, glycosides, cardiac glycerides, flavonoids, phenols, terpenoids, steroids, sterols, saponins, tannins, and phosphate	Antimicrobial	Ribka et al. (2021)
*P. grammocephalus*	Alkaloids, flavonoids, triterpenes, essential oils, phenols, fatty acids, anthraquinones, coumarins, anthrones, tannins, and steroids	Antioxidative	Aquino et al. (2018)
*A. alternata*	Not determined	Antimicrobial	Chatterjee et al. (2019)
		Antioxidative	
*Alternaria* sp	Sinapate, 4-hydroxystyrene, piceatannol, and taxifolin	Antimicrobial	Lu et al. (2020a)
		Antioxidative	
*I. obliquus*	Not determined	Antihyperglycemic	Sun et al. (2008)
		Anti-lipid peroxidative	
		Antioxidative	
*I. obliquus*	Lanosterol, 3β-hydroxy-8,24-dien-21-al, ergosterol, inotodiol, ergosterol peroxide, and trametenolic acid	Anti-inflammatory	Ma et al. (2013)
		Anticancer	
*I. obliquus*	Epicatechin-3-gallate, epigallocatechin-3-gallate, naringin, ferulic acid and gallic acid properties	Antioxidative	Xu et al. (2016)
*I. obliquus*	3β-hydroxylanosta-8,24-dien-21, (+)-fuscoporianol C, inonotsutriol E, inotodiol, inonotsutriol A, trametenolic acid, saponaceoic acid I, and chagabusone A.	Cytotoxicity	Baek et al. (2018)
		Anticancer	
*I. obliquus*	3β-hydroxy-8,24-dien-21-al, inotodiol, betulin, betulinic acid-3-O-caffeate, trametenolic acid, and melanin	Anti-inflammatory	Wold et al. (2020)
		Immunological	
		Anticancer	
*I. obliquus*	Betulin, betulinic acid, inotodiol, and trametenolic acid	Anti-proliferative	Kim et al. (2020)
		Anticancer	
*I. obliquus*	3β,22,24-trihydroxy-lanosterol-8,25-diene, oleanolic acid, 3β-hydroxy-lanoster-8,24-dien-21-acid, 3β,21-Dihydroxy-lanosterol-8,24 diene, betulin, inotodiol, 3β-Hydroxy-lanoster-8,24 dien-21-aldehyde, and lanosterol	Anti-hyperuricemic	Luo et al. (2021)
		Anti-inflammation	
*I. obliquus*	Gallic acid, ferulic acid, flavonoids epicatechin-3-gallate, epigallocatechin-3-gallate, naringin, rutin, naringenin, phelligridin G, inoscavin B, and davallialectone	Antioxidative	Zhao et al. (2021)
*I. obliquus*	Seven lanostane-type triterpenoids	Anti-neuroinflammatory	Kou et al. (2021)
*I. obliquus*	Gallic acid, protocatechuic acid, salicylic acid, vanillic acid, 2,3-dihydroxybenzaldehyde, 2,5-dihydroxyterephthalic acid, coumaric acid, caffeic acid, 4-methoxycinnamic acid, hispidin, ferrulic acid, isorhamnetin, myricetin, quercetin, syringic acid, ellagic acid, hispolon, 3,4-dihydroxybenzalacetone, and 3-O-methylellagic acid	Antioxidative	Abu-Reidah et al. (2021)
*I. obliquus*	Phelligridin D	Antioxidative	Li et al. (2021)
		Antidiabetic	
*I. obliquus*	Procyanidin, caffeic acid, p-coumaric acid, isorhamnetin-3-O-glucoside, astilbin, tangeretin, gallic acid, kaempferol, quercetin, and catechin	Antioxidative	Wang et al. (2021)
*I. obliquus*	Forty-six triterpenoids	Antihyperglycemic	Chen et al. (2021)
*I. obliquus*	Inotodiol, lanosterol, and trametenolic acid	Improved lipid accumulation	Peng et al. (2022)
*L. quercina*	Phenol and flavonoid content	Antioxidative	Ogidi et al. (2018)
*P. nuda*	Tricaproin and 13-Docosenamide, (Z)-	Antioxidative	Prasher and Manju (2019)
		Anti-tumor	
		Antibacterial	
		Immunostimulant Lipoxygenase-inhibitor	
		Anti-aging	
		Analgesic	
		Antidiabetic	
		Anti-inflammatory	
		Antidermatitic	
		Antileukemic	
		Anticancer	
		Hepatoprotective	
		Hypocholesterolemic	
		Antiulcerogenic	
		Vasodilator	
		Antispasmodic	
		Antibronchitic	
*F. flavus*	Phenolics and flavonoids	Antioxidative	Fernando et al. (2016)
*X. feejeensis*	Phenolics and flavonoids	Antioxidative	Fernando et al. (2016)
*F. torulosa*	Pentacyclic triterpenoids (fuscotorunones A and B)	Antimicrobial	Noji et al. (2021)
*G. lucidum*	Triterpenes, steroids, and polysaccharides	Antihyperglycemic	Baby et al. (2015)
*G. lucidum*	Triterpenes, steroids, and polysaccharides	Antihyperglycemic	Baby et al. (2015)
*G. applanatum*	Exopolymer	Antihyperglycemic	Yang et al. (2007)
*G. lucidum*	Lucidenic acids	Anticancer	Weng et al. (2007)
*G. lucidum*	Ethyl lucidenate (ethyl 7β-hydroxy-4,4,14α-trimethyl-3,11,15-trioxo-5α-chol-8-en-24-oate)	Antitumor	Li et al. (2013)
*G. applanatum*	Exopolysaccharides	Anticancer	Osińka-Jaroszuk et al. (2014)
		Antibacterial	
*G. lucidum*	Ganodermic acids	Anticancer	Upadhyay et al. (2014)
		Antibacterial	
*G. lucidum*	Polysaccharides	Anticancer	Li et al. (2018)
*G. sinense*			
*G. lucidum*	Lanostane triterpene, and aromatic meroterpenoids	Antioxidant	Wang et al. (2019a)
		Neuroprotective	
*G. lucidum Lurongzhi*	Ganoderic acids	Anticancer	Zhang et al. (2019)
*G. sinensi*			
*G. lucidum*	Polysaccharides	Antitumor	Bhat et al. (2021)
		Antioxidant	
		Immunomodulator	
		Antibacterial	
		Neuroprotective	
		Hypoglycemic	
		Hepatoprotective	
*G. lucidum*	Fucoxylomannan	Anticancer	Milhorini et al. (2022)
*G. applanatum*	Not determined	Antibiotic	Hassan et al. (2019)
*G. lucidum*	Polysaccharides, triterpenois	Antimicrobial	Uma Gowrie et al. (2014)
*G. lucidum*	Not determined	Antimicrobial	Hleba et al. (2014)
*G. boninense*	Dodecanoic acid, cyclododecane, octadecanoic acid, 9-octadecenoic acid, hexadecanoic acid, methyl tetradecanoate, 9, 12-octadecadienoic acid, dodecyl acrylate, and hexadecanoic acid	Antibacterial	Ismail et al. (2014)
*G. lucidum*	Not determined	Antioxidant	Hoque et al. (2015)
		Antibacterial	
		Cytotoxicity	
*G. lucidum*	Alkaloids, tannins, glycosides, and saponins	Antimicrobial	Romorosa et al. (2017)
*G. lucidum*	Triterpenoids, polysaccharides	Antiviral	Ahmad et al. (2021)
*G. boninense*	Not determined	Antibacterial	Chan and Chong (2022)
*G. neo-japonicum*	Flavonoids	Antioxidant	Ayimbila et al. (2023)
*G. lucidum*		Antibacterial	
*G. lucidum*	Polysaccharides, triterpenes, peptides, and polysaccharide peptides	Anti-aging	Wang et al. (2017)
*T. versicolor*	Not determined	Antimicrobial	Hleba et al. (2014)
*T. hirsuta*	Flavonoids	Antimicrobial	Romorosa et al. (2017)
*T. gibbosa*	Cerevisterol	Antibiotic	Appiah et al. (2020)
*T. elegans*			
*T. elegans*	Flavonoids, tannins, phenols, steroids, alkaloids, anthraquinones, anthrones, coumarins, essential oils, and fatty acids	Scavenging	Nanglihan et al. (2018)
		Antibacterial	
		Cytotoxicity	
*Trametes* spp.	Not determined	Antimicrobial	Gebreyohannes et al. (2019)
*T. versicolor*	Sequiterpenes	Antimicrobial	Gebreyohannes et al. (2019)
*T. versicolor*	Not determined	Antibiotic	Hassan et al. (2019)
*T. versicolor*	Phenolics, flavonoids, ascorbic acid, β-carotene, and lycopene	Antimicrobial	Bains and Chawla (2020)
		Anti-inflammatory	
*T. polyzona*	Caprylic acid methyl ester, tridecanoic acid methyl ester, myristoleic acid methyl ester, cis-10 pentadecanoic acid methyl ester, palmitoleic acid methyl ester, heptadecanoic acid methyl ester, stearic acid methyl ester, elaidic acid methyl ester, oleic acid methyl ester, linolelaidic acid methyl ester, g- linoleic acid methyl ester, x-linolenic acid methyl ester, heneicosanoic acid methyl ester, and cis-11-14-eicosadienoic acid methyl ester	Antimicrobial	Oyetayo and Akingbesote (2022)
*T. hirsuta*	Not determined	Antimicrobial	Begum et al. (2023)
*T. versicolor*	Sesquiterpenes	Antimicrobial	Wei et al. (2023)
*T. versicolor*	Not determined	Anti-leishmanial	Leliebre-Lara et al. (2015)
*T. versicolor*	Not determined	Anti *Fusarium langsethiae* (cereal pathogen)	Parroni et al. (2019)
*T. versicolor*	Polysaccharopeptide	Anti morphine addiction	Wang et al. (2019b)
*T. orientalis*	Polysaccharide	Chemoprotective	Zheng et al. (2017)
*T. versicolor*	Polysaccharides	Anticancer	Roca-Lema et al. (2019)
*T. versicolor*	Musarin	Anticancer	He et al. (2021)

**TABLE 2:** Illustration of chemical structures of some bioactive secondary metabolites from white-rot fungi.

Bioactive compound	Chemical structure
Sizofiran	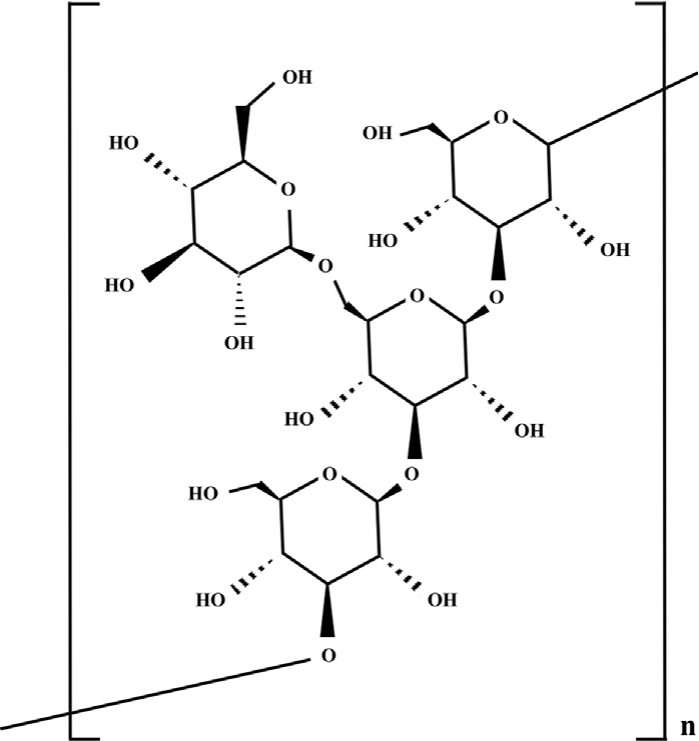
Catechin	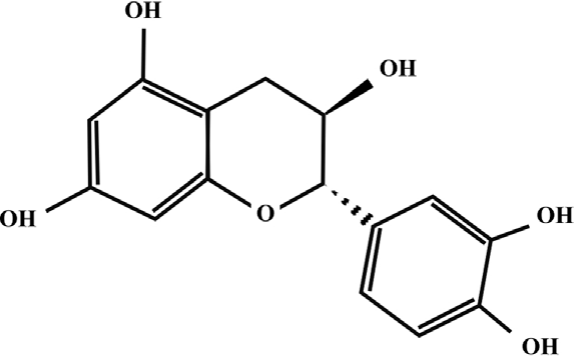
Kaempferol	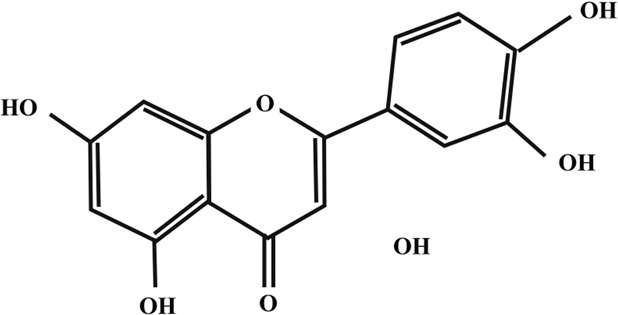
Coumaric acid	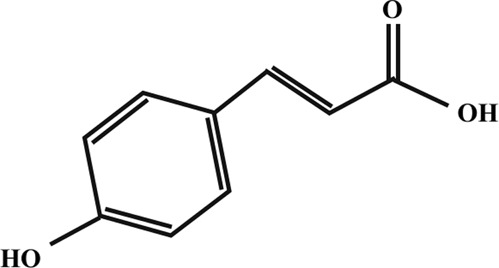
Quercetin	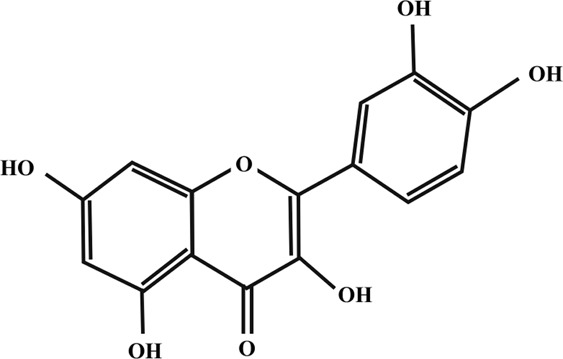
Taxifolin	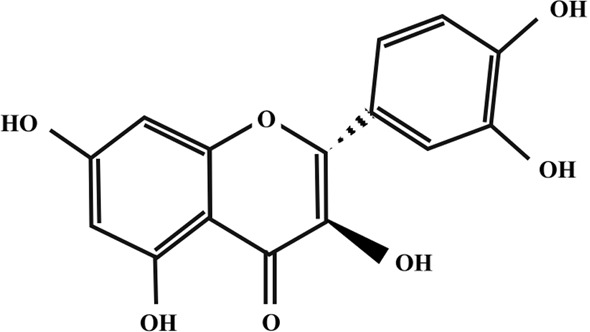
β-glucan	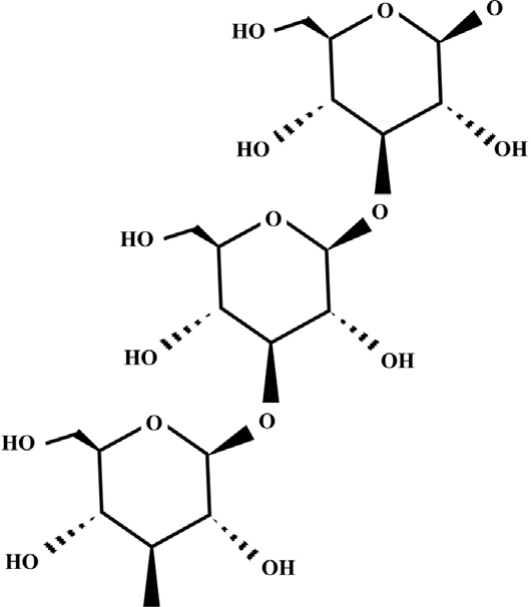
Betulin	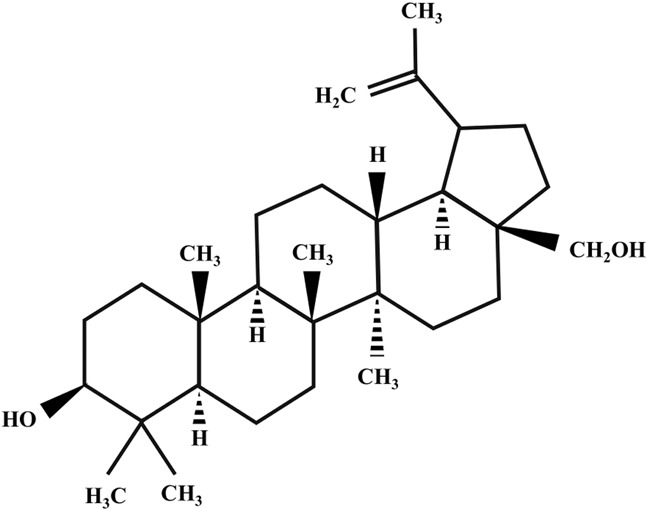
Betulinic acid	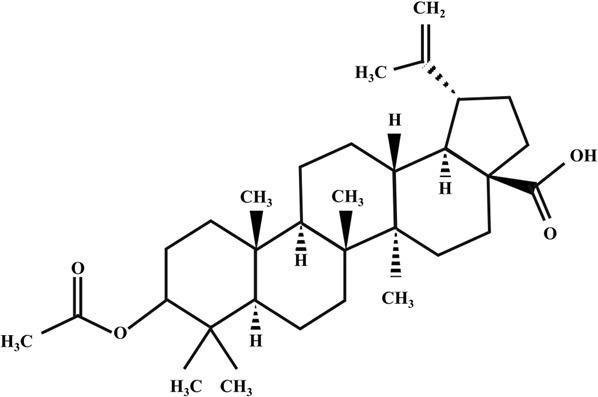
Inotodiol	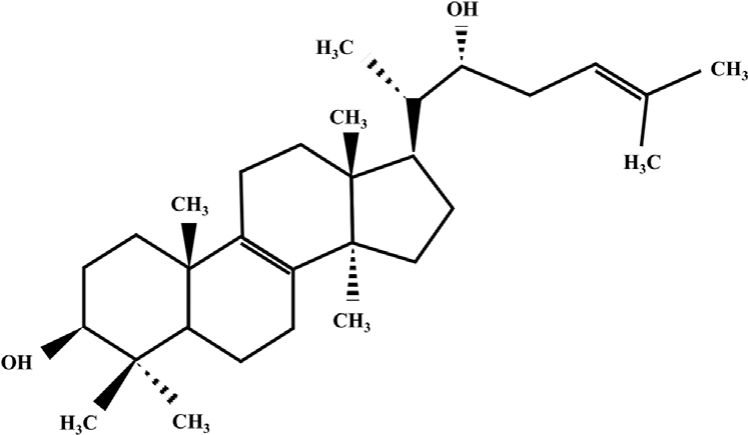
Trametenolic acid	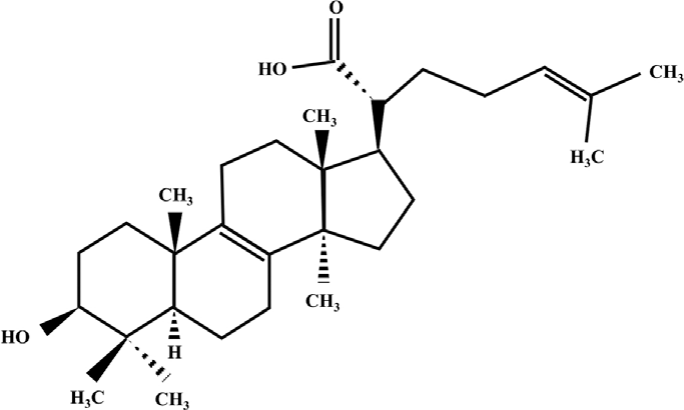
Melanin	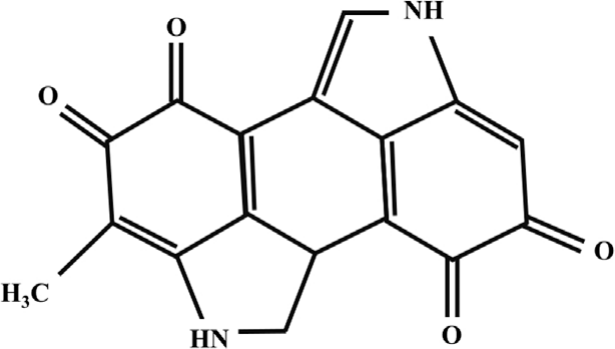
Lanosterol	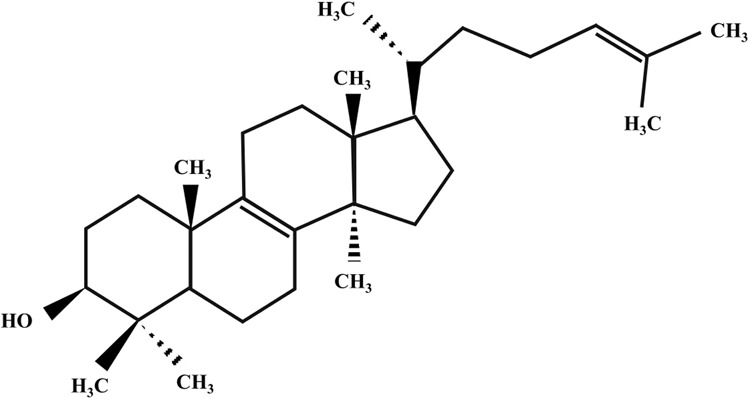
Oleanolic acid	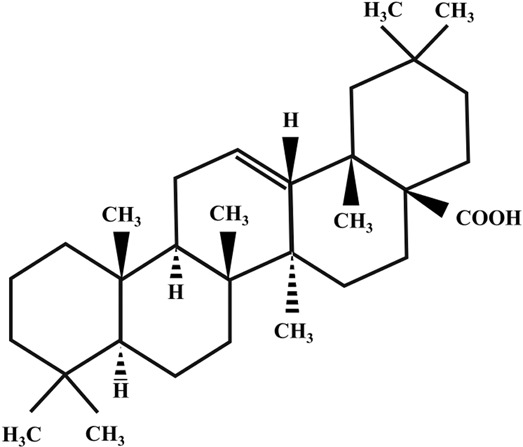
Rutin	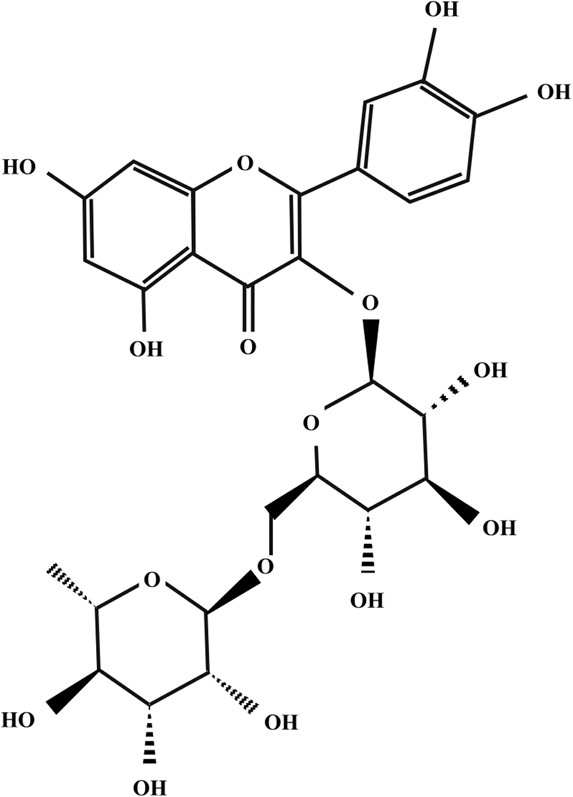

Tanimoto et al. (1996) extracted and identified a novel metabolite, schizostatin, from *S. commune* with the ability to inhibit rat liver microsomal squalene synthase to control cholesterol levels. However, in terms of antimicrobial effects, schizostatin showed no antimicrobial activity at a concentration of 1 mg/mL against many microorganisms including *Bacillus subtilis* (*B. subtilis*), *Candida albicans* (*C. albicans*)*, Escherichia coli* (*E. coli*), *Mycobacterium smegmatis*, *Mycoplasma mycoides, Proteus vulgaris*, *Proteus mirabilis*, and *Staphylococcus aureus* (*S. aureus*).

Tripathi and Tiwary (2013) investigated the production of bioactive compounds from the solvent extracts (methanol, ethanol, acetone, ethyl acetate, and hot water) of *S. commune* isolated from the Achanakmar-Amarkantak Biosphere Reserve of Central India. In that work, phenolic compounds (i.e., phenyl benzoate (C_13_H_10_O_2_) and 4-(phenyl methoxy) phenol (C_13_H_12_O_2_)) with antioxidant activity and the antibacterial compound pyrrolo (1, 2-a) piperazine-3, 6-dione (C_7_H_10_O_2_N_2_) were identified from the ethanolic and methanolic extracts, respectively. Moreover, they also detected gallic acid and L-ascorbic acid as antioxidant metabolites of both *S. commune* ethanol and methanol extracts. Therefore, it was suggested that *S. commune* could be used for the production of valuable therapeutic agents having antimicrobial and antioxidant activities.

Among several fungal isolates tested for the insecticidal potential against the tobacco cutworm *Spodoptera litura* (*S. litura*), ethyl acetate extracts of *S. commune*, isolated from Aloe vera, showed the strongest insecticidal activity (Kaur et al., 2018). The HPLC analysis of the fungal extract indicated that it contained various phenolic compounds such as gallic acid, catechin, chlorogenic acid, epicatechin, caffeic acid, coumaric acid, rutin quercetin, and kaempferol. Larvae of *S. litura* treated with that *S. commune* extract exhibited a notable decrease in the occurrence of living haemocytes with 40.00%–73.33% mortality, as well as an elevated incidence of apoptotic and necrotic cells through the cytotoxic effect of the fungal extract. Moreover, the effect of the fungal extract on tetrazolium dye mammalian viability assay (MTT) on Chinese Hamster Ovary (CHO) cell lines was carried out with a cell viability of 81.82% which was higher than the control (below 60%) consisting of doxorubicin treated cells. Additionally, the evaluation of the genotoxic effect of the *S. commune* ethyl acetate extract at various exposition times using the comet assay showed that the increasing oxidative stress triggered more DNA damage in haemocytes of *S. litura*. As a result of all the findings, the *S. commune* extract was proposed as a potential biocontrol agent.

Water, acetone, and ethanol extracts of *S. commune* exhibited a free radical scavenging activity of 19.65% at a concentration of 100 μg/mL (Kumar et al., 2018). Moreover, at the same concentration, the superoxide anion scavenging activity and the hydroxyl radical scavenging activity for *S. commune* extract was determined as 4.84% and 7.50%, respectively, whereas the total antioxidant capacity (TAC) of *S. commune* extract was found to be 12.15% using ascorbic acid as a reference standard. According to the quantitative analysis of *S. commune* mycochemicals, phenolics, flavonoids, alkaloids, tannins, and saponins were found to be 10.80 ± 0.76, 4.67 ± 0.23, 4.26 ± 0.54, 1.24 ± 0.16, and 23.83 ± 0.84 (mg/g), respectively. Compared to the edible fungi *Tricholoma nudum* and *Psalliota campestris*, a higher number of bioactive compounds (except for the content in phenolics) was obtained from *S. commune.* Overall, those results indicated that *S. commune* can serve as a valuable source of antioxidants for human health, and it was proposed that their extracts had the potential to be used for the development of drugs to lower the oxidative stress in the body.

Culture filtrate and bioactive metabolites from chloroform extracts of *S. commune* were investigated for their antimicrobial properties against different types of plant pathogens (Dutta et al., 2019). In that work, pepper fruits were treated with schizostatin and then infected with *Colletotrichum gloeosporioides* (*C. gloeosporioides*) or *Botrytis cinerea* (*B. cinerea*). For *C. gloeosporioides* infection (for anthracnose), significant effect for the control of the disease was observed from the treatment with 10 μg/mL and reached maximum with 97.8% and 100.0% by 100 μg/mL and 150 μg/mL treatment, respectively. Moreover, the control of the disease for *B. cinerea* (cause of gray mold disease) was 83.2% and 94.6% by treatment with 100 mg/mL and 150 mg/mL, respectively. The incidence of anthracnose in field conditions showed a decrease when treated with a diluted solution (12.5%) of a culture filtrate derived from *S. commune*. In that paper, the compound responsible for its antifungal and disease-control activity was identified as schizostatin. On the other hand, the growth of fungal pepper plant pathogens was inhibited by *S. commune* culture filtrate, while bacterial pathogens *Ralstonia solanacearum* and *Pectobacterium carotovorum* were unaffected by schizostatin. Thus, it was proposed that schizostatin had the potential to be utilized as a biochemical pesticide for controlling fungal infections, including anthracnose and gray mold, in various types of vegetables.

Alam et al. (2009) discovered that feeding hypercholesterolemic rats with a 5% of fruiting body powders of *Pleurotus ostreatus* (*P. ostreatus*)*, Pleurotus sajor-caju* (*P. sajor-caju*), and *Pleurotus florida* (*P. florida*) resulted in substantial reductions in total cholesterol levels (by 37.0%, 21.0%, and 16.0%, respectively) and triglyceride levels (by 45.0%, 24.0%, and 14.0%, respectively) in plasma which were attributed to the content of lovastatin in the fungal powders. Also, they compared the effect of *P. sajor-caju* on plasma and fecal lipid profiles as well as liver and kidney function in rats with high and normal cholesterol levels. The low-density lipoproteins (LDL)/high-density lipoproteins (HDL) ratio also exhibited significant decreases of 64.0%, 45.0%, and 41.0% for *P. sajor-caju*, *P. ostreatus*, and *P. florida*-fed rats, respectively. These findings based on mice studies suggested that consumption of the aforementioned *Pleurotus* species could bring notable health advantages by modulating physiological functions, particularly in addressing various atherogenic lipid profiles in cases of hypercholesterolemia, potentially serving as a nutritious source and a preventative measure against related complications and known risk factors for atherosclerosis.

Fagade and Oyelade (2009) identified and assessed 12 different species including *Auricularia auricula*, *Coriolus versicolor* (*C. versicolor*), *Daedalea elegans*, *Fomes lignosus*, *G. lucidum*, *Lentinus subnudus, Leptoporus* sp., *S. commune*, *Panus fulvus* (*P. fulvus*), *P. florida*, *Trametes saepiara*, and *Trametes betulina* for antibacterial activity. Among them, the ethanol extracts of *P. florida* and *P. fulvus* exhibited the strongest antibacterial activity against a range of bacteria including *S. aureus*, *Streptococcus* sp., *Streptococcus pyogenes* (*S. pyogenes*), *E. coli*, *Klebsiella pneumoniae* (*K. pneumoniae*), *Flavobacterium* sp., and the yeast *C. albicans* at a concentration of 1 g/mL for each microorganism. Additionally, *P. florida* displayed the lowest minimum inhibitory concentrations (MIC) value (0.01 g/mL) when tested against the yeast *C. albicans*, whereas the highest MIC was observed for *P. florida* (1 g/mL) against *Flavobacterium* sp. However, ethanolic extracts of *S. commune* and *C. versicolor* showed no inhibition against any of the tested bacteria.

The predominant bioactive component, total phenols, of the methanolic extract of *Pleurotus pulmonarius* (*P. pulmonarius*) was found to be 5.79 ± 0.03 mg/mL expressed as milligrams of gallic acid equivalent (GAE) per Gram of fruiting body (Ramesh and Pattar, 2010). The extract also contained flavonoids at a concentration of 1.76 ± 0.06 mg/mL and a minimal amount of ascorbic acid at 0.13 ± 0.00 mg/mL. The radical scavenging activity (RAS) on 2,2-diphenyl-1-picrylhydrazyl (DPPH) radical was measured at 1.62 ± 0.2 mg/mL for *P. pulmonarius*. Antimicrobial activity against a range of standard pathogenic Gram-positive and Gram-negative bacteria, along with yeast, was assessed demonstrating a notable antibacterial effect. MIC values indicated antimicrobial activity even at low concentrations (from 1 to 5 mg/mL). The methanolic extract of *P. pulmonarius* showed promising biopharmaceutical potential with its antioxidant and antimicrobial properties. However, additional research is essential to assess its effectiveness as a therapeutic compound. It is also crucial to identify the bioactive compounds and understand their mechanisms of action before considering practical applications.

Ghaly et al. (2011) evaluated the antihyperglycemic properties of an ethanol extract from *P. ostreatus* and its influence on potential DNA damage, chromosome aberrations, and sperm abnormalities in diabetic rats induced by streptozotocin. The research involved five groups of adult male albino rats, with the control group consisting of normal animals and the remaining groups comprising hyperglycemic animals. These hyperglycemic groups were orally administered with the antidiabetic drug Amaryl and different levels of mushroom extract doses: low (100 mg/kg.body weight/dL), or high (200 mg/kg.body weight/dL) for 30 days. According to their findings, the application of a higher dosage of *P. ostreatus* extract exhibited superior therapeutic effects compared to the treatment with a lower dosage. Notably, *P. ostreatus* extract, especially at high concentration, effectively lowered blood glucose levels in hyperglycemic rats, though to a lesser extent than Amaryl treatment. Significantly, mushroom treatments exhibited greater efficacy in reducing genetic alterations and sperm abnormalities in diabetic conditions compared to Amaryl treatment. In conclusion, this study highlighted the potential of *P. ostreatus* extract, particularly at higher concentration, to alleviate elevated blood glucose levels and mitigate genetic and reproductive abnormalities associated with diabetes, offering a promising alternative to conventional treatments.

Two polysaccharide fractions, namely PSPO-1a (composed of mannose, glucose, galactose, xylose, and rhamnose) and PSPO-4a (composed of rhamnose, mannose, and galactose), both containing protein and uronic acid, were isolated from ethanol extracts of *P. ostreatus* (Zhang et al., 2012). These fractions demonstrated stronger DPPH and superoxide anion radical scavenging activities, which increased with concentrations up to 2.1 mg/mL and 3.0 mg/mL, respectively. However, their efficacy in scavenging hydroxyl radicals was comparatively lower compared to DPPH and superoxide anion radical scavenging activities. Consequently, PSPO-1a proved to be a more potent free-radical scavenger than PSPO-4a which could be resulted from their different polysaccharide compositions and their varied molar ratios.

A study conducted by Mitra et al. (2013) focused on exploring the antioxidant potential and nitric oxide synthase (NOS) activation properties of water-soluble crude polysaccharides derived from *P. ostreatus*. Their results indicated that the polysaccharides, primarily composed of carbohydrates, notably β-glucan, displayed good antioxidant activity, demonstrating superiority in free radical scavenging and NOS activation compared to other components including low levels of protein and phenolic compounds. The yield from hot water extraction of dried fruit bodies revealed a total polysaccharide content of 62.67 ± 7.67 mg/100 mg, with the total glucan component as 43.9 ± 1.2 mg/100 mg. Furthermore, the polysaccharides exhibited significant NOS activation properties. Moreover, considering the antioxidant activity, the EC_50_ (the concentration required to obtain a 50% antioxidant effect) values for scavenging hydroxyl radicals, superoxide radicals, and chelating ferrous ions were 665, 390, and 370 μg/mL, respectively. That study highlighted the potential of *P. ostreatus* as a valuable source of bioactive compounds, suggesting that its crude polysaccharides, rich in β-glucan, could serve as an effective antioxidant food additive or find applications in the pharmaceutical industry.

Assis et al. (2013) investigated the antitumor activity of the exopolysaccharides (EPSs) and the mycelial biomass (intracellular polysaccharides, IPSs) of *P. sajor-caju* against Sarcoma 180 (S180) cells. According to that test, the antitumor efficacy of the produced EPS was 86%, and two IPSs from the mycelial biomass showed 80% and 82%. Since the chemical characterization of these bioactive compounds was not determined within that study, the main concern surrounding these exo- and intracellular polysaccharides lies in the lack of identification of bioactive secondary metabolites presented in EPS and IPS produced by *P. sajor-caju*. Therefore, after further investigation, that findings could aid in the exploration of new bioactive substances, introducing innovative perspectives to the medical and pharmaceutical fields.

Corrêa et al. (2015) focused on the chemical characterization and bioactivity of ethanol extracts of fruiting bodies and submerged mycelia from *Pleurotus ostreatoroseus* (*P. ostreatoroseus*). The fruiting body and mycelia extracts contained a minimum of five free sugars, four organic acids, four phenolic compounds, and two tocopherols. The culture filtrates from submerged cultivation exhibited superior reducing activity only for fruiting body (1.79 ± 0.01 mg/mL). Furthermore, DPPH scavenging activity (4.78 ± 0.02 mg/mL for fruiting body and 15.62 ± 0.13 mg/mL for mycelia), β-carotene bleaching inhibition (0.40 ± 0.01 mg/mL for fruiting body and 7.62 ± 0.25 for mycelia), and lipid peroxidation inhibition (0.29 ± 0.00 mg/mL for fruiting body and 2.34 ± 0.08 mg/mL for mycelia) in porcine (*Sus scrofa*) brain homogenates were detected in terms of bioactivity. Additionally, *P. ostreatoroseus* demonstrated higher anti-inflammatory and antimicrobial activities, while showing no hepatotoxicity in porcine liver primary cells. These functional responses could be associated with varying levels of bioactive metabolites in the fruiting body extract and the submerged culture filtrate, including phenolic acids, organic acids, and tocopherols. These bioactive compounds can be utilized to create dietary supplements for nutraceutical purposes.

*P. sajor caju*, a medicinal fungus, was reported as a notable source of secondary metabolites such as phenols (3.35 ± 0.20 mg/g), flavonoids (5.36 ± 0.31 mg/g), tannins (6.84 ± 0.12 mg/g), and alkaloids (2.81 ± 0.61 mg/g), in addition to carbohydrates, protein, amino acids, and vitamins (A, C, and E) (Devi and Krishnakumari, 2015). These secondary metabolites could have significant potential for exhibiting antimicrobial, anticancer, antipyretic, astringent, and antiviral properties. The findings in that work strongly indicated the commercial and pharmaceutical significance of the secondary bioactive compounds found in *P. sajor caju*.

Chowdhury et al. (2015) studied the antimicrobial and antioxidant properties of methanolic extracts from three edible mushrooms (*P. ostreatus, Lentinula edodes* (formerly *Lentinus edodes*) (*L. edodes*)*,* and *Hypsizigus tessulatus* (*H. tessulatus*)) isolated from Bangladesh. Antimicrobial activity against 8 microbial strains was evaluated, revealing substantial effectiveness with diameters of inhibition zone (DIZ) ranging from 7 ± 0.2 to 20 ± 0.1 mm. MIC values exhibited notable activity at concentrations ranging from 1 mg/mL to 9 mg/mL, with *L. edodes* exhibiting the most potent antimicrobial activity. *Pseudomonas aeruginosa* (*P. aeruginosa*) showed the maximum resistance, while *Saccharomyces cerevisiae* (*S. cerevisiae*) was more sensitive for the three fungal tested extracts. To reveal the antioxidant efficiency, free radical scavenging activity (EC_50,_ μg/ml) on DPPH was determined as 100 ± 1.20, 105.0 ± 1.23, and 110.0 ± 1.24 μg/mL respectively for *P. ostreatus*, *H. tessulatus*, and *L. edodes* (with ascorbic acid as a control, 5.25 ± 0.21 μg/mL). The total phenols, a major bioactive component, ranged from 3.20 ± 0.05 to 10.66 ± 0.52 mg/mL expressed as mg of GAE per Gram of fruiting bodies. Furthermore, the flavonoid concentration detected spectrophotometrically in all isolates ranged from 2.50 ± 0.008 mg/mL to 4.76 ± 0.11 mg/mL. The potential of these extracts to serve as effective therapeutic agents requires further investigation and a detailed study of their mechanisms of action prior to application.

Koutrotsios et al. (2018) cultivated *P. ostreatus*, *Pleurotus eryngii* (*P. eryngii*), and *Pleurotus nebrodensis* (*P. nebrodensis*) on unconventional substrates such as grape marc (GMC) and olive mill byproducts (OMB), with wheat straw (WHS) serving as the control. GMC-based media demonstrated comparable or superior mushroom productivity compared to WHS for *P. eryngii* and *P. nebrodensis*, while *P. eryngii* exhibited enhanced cultivation performance in OMB-based media. Both GMC and OMB substrates led to a substantial increase in the content of fruiting bodies in phenolic acids, resveratrol, triterpenic compounds, and ergosterol. Specifically, *P. eryngii* methanol extract displayed significantly high total phenolics, showing a substantial 2- to 8-fold increase in antioxidant activity based on DPPH and ferric reducing/antioxidant power assays. Moreover, substrates containing GMC or OMB resulted in up to a 27.0% increase in mushroom β-glucans. *Pleurotus* species responded differentially and mostly in a substrate-specific manner, selectively absorbing organic compounds. The phenolics and squalene content in substrates showed a strong correlation with the antioxidant activity of fungi and ergosterol, respectively. Similarly, a comparable correlation was noted between the triterpene content in substrates and fungi.

In a study by Beltrán Delgado et al. (2021), the aqueous extract of mature fruiting bodies of *P. ostreatus* exhibited higher levels of proteins, reducing sugars, and flavonoids than those in the extract of early-stage fungus. However, carbohydrates and total phenols were higher in the extract from the early stage of fungal development than in mature fruiting body extract. According to that work, the antioxidant characteristics of *P. ostreatus* aqueous extractions (earliest stage of fungal development and mature fruiting bodies) were influenced by changes in the levels of bioactive compounds, considering the physiological attributes during different growth phases. These findings could be valuable for developing protocols to obtain bioproducts from *P. ostreatus* with potential applications as antioxidants in food and medical-pharmaceutical industries and for the design and formulation of new related therapeutic products.

Ogidi et al. (2020) focused on the production of EPSsby submerged cultures of *P. pulmonarius*, containing diverse agricultural wastes. The highest EPS yield (5.60 g/L) was achieved by *P. pulmonarius* submerged cultures supplemented with groundnut shells (20.0 g/L) (EPS-B). The observed zones of inhibition by EPS-A (without agro-waste), EPS-B (groundnut shell), EPS-C (coconut husk), and EPS-D (pineapple peel) against *Shigella dysenteriae* and *E. coli* did not show significant differences. All the obtained EPS variants inhibited the growth not only of Gram-positive bacteria, including *B. subtilis* and *S. aureus,* but also against *C. albicans* and Gram-negative bacteria. All the obtained EPS exhibited DIZs (5.00–14.00 mm) against different tested microorganisms. The MIC also ranged from 0.25 to 1.00 mg/mL against the tested microorganisms. The EPS-A to D demonstrated scavenging activity within the ranges of 67.80%–81.80%, 60.60%–81.20%, 70.40%–84.70%, and 78.40%–88.50% against DPPH, OH, Fe^2+^, and NO radicals, respectively. The potential applications of the EPS, obtained from submerged cultures of *P. pulmonarius* supplemented with different agro-wastes, make it a promising natural product with the possibility of being utilized as a preservative in the food industry. Additionally, the method of generating natural bioactive compounds through fungal submerged culture using agricultural waste offers a potential solution to the unregulated disposal of agricultural waste into the environment.

Aqueous extracts from *P. ostreatus* and *L. edodes* (shiitake mushroom) exhibited the expression of 753 and 432 proteins, respectively (Elhusseiny et al., 2021). Common bioactive peptides such as Rab GDP dissociation inhibitor, superoxide dismutase, thioredoxin reductase, serine proteinase, and lectin were identified in both white rot fungal extracts Additionally, *P. ostreatus* extract contained phenolics and flavonoids, such as catechin, kaempferol, and apigenin, whereas catechin and quercetin were detected in the extract of *L. edodes.* Vitamins, including ascorbic acid, nicotinic acid, nicotinamide, and pyridoxine, together with various amino acids were also detected in both extracts. The antioxidant impact of both fungi can be ascribed to the existence of numerous bioactive elements, such as flavonoids, phenolics, bioactive peptides, and vitamin C. Notably, both extracts demonstrated significant antiviral activities, particularly *P. ostreatus* extract exhibited a selectivity index (SI) of 4.5 and 2.0 against adenovirus (Ad7) and herpes simplex virus-II, respectively, while *L. edodes* extract showed values of 2.7 and 2.5 for the respective viruses. The aqueous extracts from *L. edodes* and *P. ostreatus* demonstrated an approximately 20.0% reduction in viability among the tested cancer cell lines LS-513 (cecum carcinoma), HepG2 (hepatocellular carcinoma), DU-145 (prostate cancer), and PC-3 (prostate cancer). Cytotoxicity analysis was conducted on aqueous fungal extracts against leukemia (CCR-CEM, NB-4, THP-1) and lymphoma (U937) cells. The *L. edodes* extract exhibited a viability decrease of 66.02% in THP1 cells, while the *P. ostreatus* extract reduced the viability of CCRF-CEM cells to 70.64%. Additionally, minimal cytotoxic effects on normal human peripheral blood mononuclear cells (PBMC) from the extracts with untreated cells and doxorubicin treated cells as negative and positive controls, respectively, was observed. Considering the effects of a wide range of bioactive compounds in the aqueous extracts of the white rot fungi *P. ostreatus* and *L. edodes,* the study suggested the potential pharmacological application of these fungal strains. It underscored their minimal cytotoxicity on normal PBMCs, while also emphasizing their beneficial properties in terms of antiviral, antitumor, and antioxidant properties.

Oba et al. (2009) assessed the impact of immunochemotherapy using lentinan derived from *L. edodes* in comparison to chemotherapy alone in individuals with advanced gastric cancer through a meta-analysis of 650 individual patient data. Based on their findings, lentinan demonstrated a potentially higher efficacy in patients with lymph node metastasis in contrast to those without such metastasis. Moreover, the proportion of hepatic metastasis in the group receiving chemotherapy plus lentinan was smaller than that in the group receiving chemotherapy alone, with percentages of 34.5% and 43.1%, respectively. It was indicated that lentinan extended the overall survival period of the patients. In summary, the inclusion of lentinan alongside standard chemotherapy showed a notable and significant advantage over chemotherapy alone in terms of survival for individuals having advanced gastric cancer.

Resurreccion et al. (2016) reported the isolation of ergosterol and trilinolein from dichloromethane extracts of *L. edodes,* obtained from the Mushroom Burger in Tagaytay City, Philippines. Their structures were identified by comparing their NMR data with those of the existing literature. Ergosterol from the water extract of *Polyporus* showed significant protective properties against bladder tumor promotion in Wistar rats (Yazawa et al., 2000). Previous research also indicated that ergosterol in *P. ostreatus* extracts had the potential to inhibit lipid peroxidation (Dissanayake et al., 2009). On the other hand, trilinolein exhibited protective effects against cardiovascular disorders, including its ability to inhibit ischemia-induced ventricular arrhythmias and display antioxidant properties (Chan et al., 2002; Chan et al., 2005). In addition, trilinolein from the water extract of *Polyporus* inhibited the growth of human non-small cell lung carcinoma A549 and induce programmed cell death, with the effects being contingent on both the dosage and duration of exposure (Chou et al., 2011).

Sevindik (2018a) evaluated the antioxidant capacity of the *Lentinus tigrinus* (*L. tigrinus*) by determining the total antioxidant status (TAS), the total oxidant status (TOS), and the oxidative stress index (OSI) as 1.748 ± 0.071 mmol/L, 19.294 ± 0.237 μmol/L, and 1.106 ± 0.031, respectively. Additionally, the antimicrobial properties of ethanol, methanol, and dichloromethane extracts of *L. tigrinus* were investigated against several bacterial and yeast strains, including *S. aureus, Enterococcus faecalis* (*E. faecalis*)*, E. coli, P. aeruginosa, C. albicans, Candida krusei* (*C. krusei*) and *Candida glabrata* (*C. glabrata*) in a range from 800 to 100 MIC (µg/mL) values with highest anticandidal activity. In that paper, it was proposed that *L. tigrinus* could serve as a natural antioxidant and antimicrobial source. On the other hand, since *L. tigrinus* is an edible mushroom (Mohammadnejad et al., 2019), the restriction of over-consumption of this white-rot fungus could be necessary because of its high level of antioxidants. Moreover, the fungal extracts should be analyzed to determine the responsible bioactive secondary metabolites for its antimicrobial and antioxidant activities.

The antioxidant and antidiabetic properties of mycelium and fruiting body ethanol extracts of *Lentinus swartzii* (*L. swartzii*) was examined by Austria et al. (2021). The inhibition of α-amylase, which is an enzyme responsible for breaking down carbohydrates during digestion, has the potential to cause a decrease in blood sugar levels (Tundis et al., 2010). Considering this, the ethanolic extract of *L. swartzii* mycelium demonstrated a notable α-amylase inhibitory activity of 81.98%, while the fruiting body ethanolic extract exhibited an α-amylase inhibitory activity of 71.08%. The mycelial extract contained essential oils, triterpenes, sugars, tannins, flavonoids, fatty acids, and phenols, while the fruiting body extract presented the same components except for fatty acids and sugars. At a concentration of 1,000 μg/mL, the mycelial ethanolic extract showed scavenging effects against DPPH (35.29%) and NO (36.04%), contained 20.25 mg GAE/g sample, and demonstrated high inhibitory activity against α-amylase (81.98%). Similarly, the fruiting body ethanolic extract, at the same concentration, scavenged 43.69% of DPPH, 31.75% of nitric oxide, contained 16.92 mg GAE/g sample, and exhibited high inhibitory activity against α-amylase (71.08%). Consequently, both *L. swartzii* mycelia and fruiting body ethanolic extracts held promise as valuable sources of bioactive compounds with antioxidant and antidiabetic activities. A notable observation in that paper was that mycelia grown in coconut water exhibited superior activities compared to the fruiting body cultivated in sawdust and rice straw substrate. This signifies that the chemical properties and biological effects are impacted not just by elements like species, strain type, growth media, and solvents for extraction but also by the specific medium composition used for fungal cultivation. Further steps, including the isolation and characterization of the compounds responsible for these significant bioactivities, are crucial for a comprehensive understanding of the extracts’ potential in different applications.

Muslihin et al. (2022) reported that the wild mushroom *Lentinus squarrosulus* (*L. squarrosulus*) possessed notable characteristics such as rapid mycelium growth, having potential to be a food source, and various other benefits. Notably, it serves as a source of bioactive compounds. The ethyl acetate extract from *L. squarrosulus* was analyzed at 516.8 nm using a UV-Vis spectrophotometer and it revealed a strong antioxidant activity with an EC_50_ of 54.93 mg/L. This highlights its possible utility in various applications.

Lin et al. (2008) investigated the impact of *Lycium barbarum* (*L. barbarum*) fruit extract on the growth and extracellular polysaccharopeptide (ePSP) production by *C. versicolor* (now *Trametes versicolor* (*T. versicolor*)) in a 20-L fermenter under submerged fermentation conditions. The addition of *L. barbarum* extract (LBE) into the culture medium led to a notable increase in ePSP production as from 0.61 g/L to 1.66 g/L. Significantly, ePSP from *C. versicolor* cultured with supplemental *L. barbarum* extract demonstrated noteworthy immunomodulatory activity, influencing the production of nitric oxide and various cytokines by murine RAW264.7 cells. The approach in that work can open up new possibilities for the future advancement of dietary supplements centered around *C. versicolor* LH1 polysaccharopeptides.

From a submerged culture of *Panus strigellus* (*P. strigellus*), Llanos-López et al. (2023) isolated three metabolites, including a new bioactive compound called panapophenanthrin and two known compounds identified as panepophenanthrin and dihydrohypnophilin which are defined in an uncommon group of oligocyclic terpenoidal metabolites, exclusively identified in the *Panus* genus. While panapophenanthrin and dihydrohypnophilin exhibited not a very strong antimicrobial effect with MIC ranging from 33.3 to 66.6 g/mL on various fungal strains together with Gram-positive and Gram-negative bacteria, panepophenanthrin showed no activity against any of the tested microorganisms. Moreover, panapophenanthrin showed strong cytotoxic effects on mammalian cell lines including mouse fibroblast (L929) and human endocervical adenocarcinoma (KB3.1) with 13.2 and 17.9 EC_50_ (µM) values, respectively. *Panus* species are predominantly found in tropical and subtropical areas. Hence, this discovery emphasized the significance of examining tropical species to uncover new bioactive compounds, but additional research is necessary to thoroughly understand the bioactivity of these compounds and explored their potential uses in different applications.

*Cyclocybe cylindracea* (*C. cylindracea*) ethanol extract was investigated to determine its phenolic content, heavy metal content, and antioxidant activity to evaluate possible medical benefits (Sevindik et al., 2018). In that work, TAS, TOS, and OSI values were determined as 4.325 mmol/L, 21.109 μmol/L, and 0.488. Phenolic compounds including gallic acid, hesperidin, catechin, syringic acid, and hydroxybenzoic acid, were detected in the ethanolic extracts of *C. cylindracea*. These bioactive compounds presented diverse health advantages, encompassing antioxidant properties, anti-inflammatory effects, and potential anti-cancer properties (Sevindik et al., 2018). Despite possible benefits, since *C. cylindracea* is edible (Landingin et al., 2020), its levels of Pb (16.54 ± 0.93 mg/kg) and TOS values should be considered.

Masuda et al. (2008) explored the antimetastatic properties of *Grifola frondosa* (*G. frondosa*) (maitake mushroom) extracts*,* using an experimental mice model of lung metastasis. The observed inhibition of lung metastasis by *G. frondosa* extract was attributed to the activation of NK cells. Additionally, the *G. frondosa* extract demonstrated inhibition of ICAM-1 (Intercellular Adhesion Molecule 1) expression in vascular endothelial cells, suggesting that its mechanism of action involved blocking the adhesion of tumor cells to lung tissue, thereby inhibiting metastasis. Their findings suggested that *G. frondosa* extract was effective for cancer prevention and the inhibition of tumor metastasis when consumed regularly.

Masuda et al. (2009) explored the ability of an extract from *G. frondosa* to enhance the immune system by antitumor and antimetastatic activities together with cisplatin, a well-known anticancer drug. Based on their findings, the increased antitumor and antimetastatic effectiveness observed in the combination of cisplatin with *G. frondosa* extract was attributed to a synergistic interaction. This synergy was sourced from the dual action of cisplatin’s cytotoxic impact on tumor cells and the simultaneous activation of the immune response in antigen-presenting cells (APC) and natural killer (NK) cells by *G. frondosa* extract. Moreover, this combination not only exhibited antitumor and antimetastatic activity but also caused a decrease in cisplatin-induced myelotoxicity and nephrotoxicity. Consequently, the joint administration of *G. frondosa* extract with cisplatin holds promise as a beneficial approach to cancer treatment.

The clinical evaluation of the immunological effects of hot water and alcohol extract from the fruit body of *G. frondosa* at different oral dosage levels was first investigated for a group of 34 eligible study subjects in the work performed by Deng et al. (2009). Based on their results, it was shown that the administration of *G. frondosa* extract was linked to notable alterations in specific immunologic parameters within the peripheral blood. According to their findings, this extract was recognized for its role as an immunomodulator rather than simply an immune enhancer. Moreover, cancer patients should be aware of that *G. frondosa* extracts may have complex effects on immune function, and while the clinical impact on cancer prevention or treatment remains uncertain, it is crucial to conduct experimental investigations to clarify its potential anticancer effects.

Su et al. (2020) prepared a *G. frondosa* extract through a process involving hot water extraction from the fruiting body, followed by enzymatic digestion and dialysis, resulting in high and low molecular weight fractions. They examined the water-soluble polysaccharides of *G. frondosa* to understand their impact on inflammation and receptor interactions using parental RAW264.7 macrophages and Dectin-1-expressing RAW264.7 macrophages. The results of cell-based assays indicated that the high molecular weight fraction (1,260 kDa) as the major bioactive fraction demonstrated inhibitory effects on tumor necrosis factor-α (TNF-α) and interleukin-6 (IL-6) production, while also reducing NF-κB (important transcription factor regulating inflammatory responses in eukaryotes) activation in lipopolysaccharide-induced macrophages. That research suggested that the nondigestible β-(1→6)-branched (1→4)-β-D-glucan found in high molecular weight fraction might be responsible for its anti-inflammatory properties by interacting with TLR2 receptors rather than Dectin-1 or CR3 receptors. The discovered polysaccharide was identified as a non-digestible glucan with a β-linked core and side groups. Moreover, the receptor-binding properties and anti-inflammatory activity of *G. frondosa* polysaccharides may be influenced by their molecular weight and arrangement of linkages.

Eckhardt et al. (2000) reported the impact of a bioactive fungal compound on pancreatic cancer in humans. This involved a phase I trial and a pharmacokinetic examination of irofulven (a fungal cytotoxin) (doses ranging from 1.0 to 17.69 mg/m^2^) and a novel cytotoxin derived from the white-rot fungus *Omphalotus olearius* (*O. olearius*), which was conducted on 46 patients (given daily for 5 consecutive days every 4 weeks) with advanced solid malignancies. According to the trial, evidence of antitumor activity was observed in an individual with advanced pancreatic cancer, coupled with the remarkable preclinical antitumor effects demonstrated by irofulven.

Chandra et al. (2019) assessed the antioxidant activity of the white-rot fungi *Phanerochaete chrysosporium* (*P. chrysosporium*), *Phlebia brevispora* (*P. brevispora*), and *Phlebia floridensis* (*P. floridensis*) against various free radicals, including DPPH, nitric oxide, ferrous ion, and ferric ion, in addition to their total phenolic content. All the studied fungal strains produced phenolics ranging from 5.2 to 16.7 mg/mL and exhibited diverse free radical and metal ion scavenging activities. The growth medium significantly influenced these activities. Thus, all the studied fungi presented similar antioxidant activity (approximately 72.0% DPPH scavenging) in yeast extract glucose medium, while it was lower in Czapek dox’s medium (ranging from 60.0% to 45.0%). The fungal extracts showed no mutagenic or cytotoxic effects, highlighting the fungi’s potential as a new source for the rapid production of extracellular antioxidants. These white rot fungi displayed strong antioxidant potential and could serve as a valuable source of natural antioxidant compounds. Further studies are recommended to isolate and characterize the bioactive compounds for potential use in new therapeutic approaches.

*Hericium erinaceus* (*H. erinaceus*) is a well-known traditional medicinal fungus acclaimed for its anti-dementia properties, Alzheimer’s disease, depression, Parkinson’s disease, and spinal cord injuries together with the production of cyathane diterpenoids (erinacines). Numerous structurally diverse bioactive compounds, exceeding 80 types, have been extracted from both the fruiting bodies and mycelia of *H. erinaceus*. These compounds, including terpenoids (erinacines), phenols (hericenone AE), pyrones (erinapyrones AC), sterols (erinarol, hericerins, hericenes), fatty acids, and non-ribosomal peptides, exhibit various therapeutic effects such as anti-tumor, antibacterial, hypoglycemic, and neuroprotective activities. Around 20 of the 25 isolated diterpenoids extracted from *H. erinaceus* share a characteristic 5-6-7 tricarbocyclic fused core structure (Kawagishi et al., 1992; Kawagishi et al., 1996; Lee et al., 2000; Li et al., 2001; Kenmoku et al., 2002; Kawagishi et al., 2006; Kobayashi et al., 2014; Lu et al., 2014; Friedman, 2015; Li et al., 2015; Wang et al., 2015; Brandalise et al., 2023).

Lee et al. (2014) reported that erinacine A from the ethanolic extract of *H. erinaceus* was able to inhibit inflammatory cytokine expression as a neuroprotective effect in adult male Sprague–Dawley rats having ischemia injury. In that work, it was also shown that the brain tissue trauma effectors of nitrotyrosine (RNS) via inducible nitric oxide synthase (iNOS) (Alderton et al., 2001)/p38 mitogen-activated protein kinase (MAPK) (Darling and Cook, 2014; Han et al., 2020)/a transcription factor (i.e., CHOP) (Bruhat et al., 1997) contributed to the neuroprotective effect. Furthermore, in a stroke animal model, erinacine A led to the suppression of reactive nitrogen species and the downregulation of iNOS, p38 MAPK, and CHOP, which are factors involved in ischemia injury.

*H. ernaceus* methanolic extracts inhibited the inflammatory activity induced by lipopolysaccharide/interferon-γ in murine RAW264.7 cells, with a maximum decrease in nitric oxide production of 39.6% (Lee et al., 2016). The bioactive metabolites in these methanol extracts were identified as three different hericenones, C, D, and F. Consequently, they suggested that the anti-inflammatory effect of the *H. ernaceus* extract was likely based on the hericenones F. Also, ethanolic extracts of *H. erinaceus* myceliaeffectively inhibited glutamate-induced apoptosis in PC12 cells against 20 mM glutamate-induced damage (Chang et al., 2016). The following biochemical parameters glutathione at 2.5 ± 0.7 nmol/mg protein, glutathione peroxidase at 28.2 ± 3.2 mU/mg protein, glutathione reductase at 2.3 ± 0.4 mU/mg protein, calcium influx at 360 ± 23 nmol/L, reactive oxygen species at 140.0% ± 7.0%, superoxide dismutase at 23.2 ± 4.2 U/mg protein, H_2_O_2_ at 20.4 ± 3.5 nmol/mg protein, and thiobarbituric acid reactive substances (malondialdehyde) at 13.3 ± 2.5 mmol/mg protein, were affected by glutamate insult. Overall, their findings underlined the potential neuroprotective effect of erinacine A from *H. erinaceus* ethanolic extracts.

Despite the elucidation of chemical synthesis, the biosynthetic pathway and gene regulation remain unknown. A comparative genome analysis of 42 basidiomycota fungal species, including *H. erinaceus*, revealed abundant gene clusters related to terpenoid and polyketide biosynthesis (Chen et al., 2017). The genome analysis of *H. erinaceus* will provide important understanding into the biosynthetic pathways of bioactive secondary compounds, which is crucial for improving the production of these compounds.

Zhang et al. (2017) explored the neuroprotective and neuritogenic properties of several secondary metabolites, including 4-chloro-3,5-dimethoxybenzoic methyl ester, 3-(hydroxymethyl)-2-furaldehyde, erinacine A, erinacerin G, herierin III, and herierin IV from the methanol extract of *H. erinaceus* mycelium. Among them, 4-chloro-3,5-dimethoxybenzoic methyl ester and erinacine A metabolites not only enhanced nerve growth factor-induced neurite outgrowth but also protected neuronally differentiated cells against lack of nerve growth factor in PC12 pheochromocytoma cells. Erinacine A additionally stimulated neuritogenesis in primary rat cortex neurons. Their findings suggest that *H. erinaceus* holds promise as a potential therapeutic agent for reducing the risk of various neurodegenerative diseases.

Erinacine A and S, isolated from of *H. erinaceus* mycelia, displayed anti-neurodegenerative and neuroprotective effects in the cerebrum of transgenic mice (Tzeng et al., 2018). Thus, 30-days application of erinacine A and S attenuated cerebral plaque loading by inhibiting plaque growth, diminishing glial cell activation, and promoting hippocampal neurogenesis in transgenic mice as Alzheimer’s disease model. Additionally, it was showed that erinacine A recovered behavioral deficits in transgenic mice. These findings suggested the possibility that erinacine A may have therapeutic potential for treating Alzheimer’s disease.

Ratto et al. (2019) reported that ethanol extract of *H. erinaceus*, containing erinacine A, hericenone C, and hericenone D bioactive metabolites, was able to partially revert the cognitive and locomotor frailty index during physiological aging in a mice model. They observed an increase in proliferating cell nuclear antigen (PCNA) and doublecortin (DCX) levels in the hippocampus and cerebellum of mice supplemented (2 months) with *H. erinaceus* extract orally, indicating the occurrence of neurogenesis in elderly frail mice. Therefore, it was demonstrated that the supplementation of *H. erinaceus* extract reversed the age-related decline in recognition memory.

Roda et al. (2021) demonstrated that a two-month oral supplementation of ethanol extract from *H. erinaceus*, which contained erinacine A, hericenone C, hericenone D, and ergothioneine, could reverse age-induced cerebellar alterations in C57BL-6J wild-type male mice. These alterations included volume reduction, molecular layer thickness decrease, and dwindled neurons. Additionally, the supplementation led to a decrease in inflammation, oxidative stress, and reactive gliosis. In another study, they investigated the preventive effects of *H. erinaceus* ethanol extract, which contained a high amount of ergothioneine, on cognitive and locomotor decline during physiological aging in C57BL-6J mice. The ergothioneine-rich extract exhibited neuroprotective and preventive actions, mitigating age-dependent deficiencies (Roda et al., 2022). Moreover, same extract was shown to reduce oxidative stress and inflammation in the hippocampus, prevent recognition memory decline, and increase the expression of specific receptors crucially involved in glutamatergic neurotransmission in the same mice (Roda et al., 2023).

A tremulane sesquiterpene, named irpexlacte A (yellowish needle crystals), along with three novel furan derivatives, identified as irpexlacte B (yellowish oil), irpexlacte C (yellowish powder), irpexlacte D (brown flaky solid), were obtained from the fungus *Irpex lacteus* (*I. lacteus*) isolated from waterlogging tolerant plant *Distylium chinense* by Duan et al. (2019). Furthermore, they also isolated two known metabolites, irlactin E and 3β-hydroxycinnamolide. Irpexlacte A and D demonstrated robust antioxidant activity, with EC_50_ values of 2.50 and 5.75 μM, respectively. Moreover, in contrast to gentamicin (0.18 μM) as the positive control, four new compounds, irpexlacte A, B, C and D, demonstrated moderate activity, displaying MIC values of 24.1, 32.3, 35.5, and 23.8 μM, respectively, against *P. aeruginosa*. On the other hand, the isolated compounds showed no activity against tested cancer cell lines. However, irpexlacte A-D displayed significant antioxidant activity, underscoring the need for further investigations to evaluate their importance and clarify underlying mechanisms.

*Porodaedalea pini* (*P. pini*) has been an esteemed traditional mushroom known for its therapeutic properties against various diseases. In this context, Devi et al. (2022) determined the antioxidant potential of hexane, chloroform, ethyl acetate, and methanol extracts of *P. pini* using DPPH assay (EC_50_, 253.98 μg/mL, maximum with hexane extraction), total antioxidant capacity (231.04 ± 1.75 μg ascorbic acid equivalents/g of dried extract, maximum with methanol extraction), total phenolic content (277.67 ± 9.46 μg GAE/g of the sample, maximum with methanol extraction), and total flavonoid content (4.95 ± .013 μg rutin equivalent/g of dried extract, maximum with methanol extraction). The presence of 12 polyphenolic metabolites, including gallic acid, catechin, chlorogenic acid, epicatechin, caffeic acid, umbelliferone, coumaric acid, tert-butyl-hydroquinone, and quercetin was revealed. However, rutin, elagic acid, and kaempferol were not detected. The identified polyphenols of *P. pini*. could potentially contribute to its antioxidant activity, Moreover, further exploration of *P. pini* extracts is necessary to unveil its nutraceutical and pharmacological potential.

Ildız et al. (2022) employed molecular techniques to analyze the total phenolic compound content, antioxidant activity using the DPPH scavenging method, and antimicrobial activity for *Bjerkandera adusta* (*B. adusta*). Ethanol and methanol were used for extraction and the methanolic extract of *B. adusta* exhibited a total phenolic content of 772.28 μg GAE/mL. The ethanol extract demonstrated a substantial 79.66% scavenging activity against a 0.1 mM DPPH solution. For antimicrobial activity, the ethanolic extract exhibited significant antimicrobial activity, showing the largest DIZ of 28 ± 1 mm against *P. aeruginosa*. In contrast, the methanol extract displayed the lowest antimicrobial efficacy, with a DIZ of 8.7 ± 1.2 mm against *Salmonella typhimurium* (*S. typhimurium*). These findings suggested that both ethanolic and methanolic extracts of *B. adusta* possess antioxidant and antibacterial properties. More comprehensive investigations into wild-collected fungal strains should be done for an extensive exploration of the bioactive constituents present in fungi, drawing attention to their potential applications in the development of functional foods and other potential uses.

The antioxidant and oxidant potentials of the ethanolic extracts of *Hohenbuehelia myxotricha* (*H. myxotricha*) was determined for the first time by Krupodorova et al. (2022). The highest recorded TAS, TOS, and OSI values for *H. myxotricha* were 5.416 ± 0.150 mmol/L, 1.320 ± 0.156 μmol/L, and 0.024 ± 0.003, respectively. The ethanolic extracts of *H. myxotricha* exhibited antimicrobial activities with concentrations ranging from 25 to 200 μg/mL against various bacteria and yeasts. The extract demonstrated a better antifungal activity compared to its antibacterial activity. The antioxidant, oxidant, and antimicrobial potentials of *H. myxotricha* mycelia exhibited variations based on the culture media employed. According to their findings, glucose peptone yeast (GPY) medium was found more suitable for the synthesis of antibacterial bioactive metabolites against *E. coli*, while Sabouraud dextrose broth (SDB) medium was more proper for the production of antioxidant and antifungal bioactive metabolites. Thus, their findings underscored the importance of identifying an optimal cultivation medium to maximize antimicrobial and antioxidant activities. Overall, the ethanolic extract of *H. myxotricha* mycelia presented significant pharmacological potential, serving as a natural source of antioxidants and antimicrobials with potential health benefits. Like various studies in the literature, further research is required to isolate and identify the bioactive secondary metabolites responsible for the observed antioxidant and antimicrobial effects, offering potential sources for pharmacological drug designs.

Jaszek et al. (2013) isolated bioactive compounds (crude endopolysaccharides - c-EPL, and low molecular secondary metabolites - ex-LMS) extracted from *Cerrena unicolor* (*C. unicolor*) submerged cultures that exhibited antioxidant and antibacterial properties., Ex-LMS demonstrated the highest antioxidant capability (39.0%–90.0% for chemiluminometric measurement, 20.0%–90.0% for ABTS, and 10.0%–59.0% for DPPH reduction at 6.25–800 μg/mL). Moreover, c-EPL scavenging abilities ranged from 36.0% to 70.0% for chemiluminometric measurement, 2%–60% for ABTS, and 28.0%–32.0% for DPPH reduction at 6.25–800 μg/mL. Preliminary data for the toxic effect against *Vibrio fischeri* (*V. fischeri*) were found to be 85.37% for c-EPL, and 99.8% for ex-LMS. In this sense, c-EPL showed antibacterial activity against *S. aureus* with an 18.96 ± 0.4 mm DIZ while ex-LMS displayed activity with 11.83 ± 0.2 and 25.86 ± 0.2 mm DIZs, respectively, against *E. coli and S. aureus*. These compounds have the potential to serve as a novel and easily producible source of effective antioxidants within laboratory-scale conditions. Additionally, further investigation of the aforementioned bioactive secondary metabolites is crucial in terms of applications, as they may play a critical role in new therapies and serve as a natural source of antioxidative molecules.

Mizerska-Dudka et al. (2015) explored the antiviral, immunostimulatory, cytotoxic, and antitumor effects of bioactive compounds from *C. unicolor*, specifically endopolysaccharides (c-EPL) and an extracellular low molecular weight compound (ex-LMS) obtained from the culture filtrate below 10 kDa. The study employed THP-1-derived macrophages to assess immunomodulatory activity, revealing that the fungal c-EPL stimulated the production and secretion of TNF-α and IL-6. Antitumor activity was evaluated using cervical carcinoma cell lines SiHa and CaSki, with SiHa showing cytotoxic EC_50_ (µg/mL) value of 1.2 for ex-LM, and CaSki values of 2.3 for ex-LMS. The research highlighted the promising immunomodulatory effect of c-EPL samples and the need for further investigations into these multifaceted bioactive compounds.

The antioxidant and antimicrobial properties of ethanol, methanol, and dichloromethane extracts of *C*. *unicolor* were studied by Sevindik (2018b). Considering antioxidant effects, TSS, TOS, and OSI were measured as 6.706 ± 0.059 mmol/L, 19.308 ± 0.114 μmol/L, and 0.288 ± 0.003. Additionally, all the extracts presented antimicrobial efficacy within the concentration range of 25–400 μg/mL, spanning a spectrum of MIC values from 400 to 50 μg/mL against S*. aureus, E. faecalis, E. coli, P. aeruginosa, Acinetobacter baumannii, C. albicans, C. glabrata*, and *C. krusei* with higher anticandidal activity. The primary issue about these extracts is their unidentified contents regarding the bioactive secondary metabolites.

Matuszewska et al. (2019) explored the anticancer and antioxidant properties of low molecular weight secondary metabolites produced by *C. unicolor.* These secondary metabolites consisted of protein, sugars, and phenolic compounds. The findings revealed that the low molecular weight compounds displayed inhibitory effects on human colon cancer cells HT-29 within the concentration range of 25–200 μg/mL and demonstrated dose-dependent inhibition of cell proliferation, ranging from 47.5% to 9.2% at the highest concentrations. Microscopic observations indicated that all compounds induced programmed cell death, specifically apoptosis (up to 44.4% for a compound in HT-29 and less than 20.0% for most compounds in CCD 841 CoTr), with minimal or significantly low levels of necrosis observed in both cell lines simultaneously.

Romorosa et al. (2017) found that distilled water, aqueous, and acetonitrile extracts of *Auricularia fuscosuccinea* (*A. fuscosuccinea*) fruiting bodies contained alkaloids and tannins glycosides, while saponins and flavonoids were absent. The antibacterial properties of the aqueous and acetonitrile extracts were evaluated against *S. aureus* and *E. coli*. The results indicated low antibacterial activity against *S. aureus* for all the fungal extracts with DIZs of 5.0 mm, 13.5 mm, and 5.0 mm, respectively, compared to cotrimoxazole (control) with a 33.54 mm DIZ. For *E. coli*, the corresponding DIZs were 5.0 mm, 22.98 mm, and 22.41 mm, which were lower than the control with a 32.00 mm zone.

Kalaw and Albinto (2014) evaluated the antibacterial properties, phytochemical composition, and antioxidant activity of ethanol and acetone extracts of *Coprinus comatus* (*C. comatus*) and *Pleurotus cystidiosus* (*P. cystidiosus*). Both ethanol and acetone extracts exhibited antibacterial activity against *S. aureus*. The ethanol extract of *C. comatus* displayed a slightly larger DIZ (14.09 ± 4.65 mm) compared to the acetone extract (13.16 ± 3.39 mm). Conversely, in *P. cystidiosus*, the acetone extract exhibited a larger DIZ (15.25 ± 2.76 mm) than the ethanol extract (13.43 ± 0.15 mm). There was no inhibition against *E. coli* for both fungal extracts. Moreover, phytochemical screening of the extracts revealed the presence of alkaloids, flavonoids, saponins, and terpenoids in both fungal species. Steroids and cardiac glycosides were absent in *P. cystidiosus* while tannins were not detected in any of the studied species. *P. cystidiosus* registered higher DPPH radical scavenging activity (72.97% ± 0.68% to 66.59% ± 0.83%) indicating its potential antioxidant capacity, and lower total phenolic content (3.41 ± 0.12 mg GAE/g) than *C. comatus* (17.82 ± 0.51 mg GAE/g).

In a research conducted by Stilinović et al. (2020), it was reported that *C. comatus* methanol extract contained significant amounts of proteins (23.07 ± 0.28 g/100 g dry matter), carbohydrates (40.42 ± 0.48 g/100 g dry matter), dietary fibers (21.13 ± 0.34 g/100 g dry matter) and fats (2.04 ± 0.03 g/100 g dry matter). Furthermore, methanol extract of *C. comatus* served as a valuable flavonoid content (0.39 ± 0.08 (mg quercetin equivalents (QE)/g dry weight extract) and a total phenolic source (107.02 ± 2.42 mg GAE/g dry weight extract) including 4-hydroxybenzoic acid, protocatechuic acid, cinnamic acid, p-coumaric acid, caffeic acid, and quinic acid with concentrations of 11.41 ± 1.17, 0.13 ± 0.03, 4.34 ± 0.27, 10.48 ± 0.94, 0.15 ± 0.02, and 9.10 ± 1.39 μg/g, respectively, based on spectrometric analysis. According to the findings of experiments involving rats having liver damage induced by carbon tetrachloride, the administration of *C. comatus* orally for 42 days exhibited hepatoprotective effects in oxidative stress-induced liver damage by triggering repair mechanisms. Based on the results indicated in that paper, *C. comatus* had the potential to be utilized as a readily available food source with high levels of natural antioxidants. It also can be used as an additive or component for producing nutraceuticals and functional foods. Considering the reported work, an important issue arises regarding the complex composition of *C. comatus* extracts. Additional investigation is needed to ascertain whether the positive effects result from a singular active compound, or the synergistic activities of various metabolites present in the extract.

*Phylloporia ribis* (*P. ribis*), traditionally used in China for natural medicine, is recognized for its functional ingredients beneficial in treating conditions like pharyngitis, laryngitis, tonsillitis, and hyperglycemia. Ribka et al. (2021) reported bioactive compounds, and antifungal activity of methanolic (from 5% to 25%) extracts of *P. ribis*. Such extracts contained a diverse array of bioactive compounds, including carbohydrates, proteins, amino acids, lipids, alkaloids, glycosides, cardiac glycerides, flavonoids, phenols, terpenoids, steroids, sterols, saponins, tannins, and phosphate. The methanolic extract of *P. ribis* presented superior antifungal activity, particularly against *Aspergillus niger* (*A. niger*)*,* causing soft rot in carrots, with 100% inhibition observed in all methanolic extracts except at 5% concentration. The diverse components present in *P. ribis* hold promise for applications as immunity boosters, food supplements, and in the field of drug discovery. Further investigations are also required to isolate the bioactive compounds responsible for immunity-boosting, drug development, antioxidant, anti-inflammatory, antibiotic, and antimicrobial activities in their pure form.

*Polyporus grammocephalus* (*P. grammocephalus*) ethanol extract was studied for its nutraceutical potential considering its bioactive metabolites. Aquino et al. (2018) identified sugars, alkaloids, flavonoids, triterpenes, essential oils, phenols, fatty acids, anthraquinones, coumarins, anthrones, tannins, and steroids, while terpenoids, cardiac glycosides, whereas saponins were not present in the *P. grammocephalus* ethanol extract. The ethanol extract of *P. grammocephalus* displayed DPPH radical scavenging activity (26.37%) and total phenolic content of 38.58 mg GAE/g. Brine shrimp toxicity assay indicated high toxicity with an LC_50_ value of 73.78 μg/mL These findings suggested that *P. grammocephalus* extract was rich in bioactive compounds with significant pharmacological activities, including antioxidant properties and cytotoxic effects.

The antioxidant and antimicrobial properties of ethyl acetate extracts from *Alternaria alternata* (*A. alternata*) were investigated by Chatterjee et al. (2019). The ethyl acetate extracts showed MIC ranging from 300 to 400 μg/mL against both Gram-positive and Gram-negative bacteria. Moreover, the ethyl acetate extract of *A. alternata* displayed antibacterial inhibition on *B. subtilis, Listeria monocytogenes, S. aureus, E. coli, and S. typhimurium* with up to 14 ± 1.5 mm DIZ. Furthermore, a reduction in the activity of key metabolic pathways, including the EMP pathway, TCA cycle, and gluconeogenic enzymes, suggested interference with the central carbohydrate metabolism. Additionally, *A. alternata* extract demonstrated strong antioxidant potential through DPPH and superoxide radical scavenging assays, with EC_50_ values of 38.0 ± 1.7 μg/mL and 11.38 ± 1.2 μg/mL, respectively. Within this analysis, ascorbic acid, used as a positive control, had an EC_50_ value of 20.23 ± 2.3 μg/mL. These results suggest that *A. alternata* showed potential as a source of bioactive compounds with medicinal importance, demonstrating strong antibacterial effects.

Phenylpropanoid (PPPN) compounds are widely utilized in various industries due to their diverse bioactivities, including applications in agriculture, medicine, food, and cosmetics. In this sense, *Alternaria* sp., which is, a novel natural source of PPPNs, was isolated from grapes by Lu J. et al. (2020). However, starvation is known to stimulate the PPPN pathway in plants, its impact on fungi remains underexplored. In that study, metabolomics analysis revealed that starvation treatment significantly increased the accumulation of shikimate and PPPN compounds in *Alternaria* sp. Notably, the study also identified additional PPPNs, such as sinapate, 4-hydroxystyrene, piceatannol, and taxifolin, under starvation conditions. These findings indicated that starvation treatment offers an effective strategy to enhance PPPN production and unveil compounds undetectable under non-starvation conditions. Overall, subjecting *Alternaria* sp. to starvation treatment during cultivation resulted in the robust activation of both the shikimate and PPPN pathways. These findings can shed light on the potential for optimizing the production of PPPN compounds by fungi, offering insights into the genetic resources and secondary metabolite pathways of *Alternaria* sp. for future functional studies.

*Inonotus obliquus* (*I. obliquus*)*,* naturally grows on the trunks of birch wood trees in colder northern climates and is a medicinal fungus that has been used for therapeutic purposes since the 16th century (Ern et al., 2023). To investigate the antihyperglycemic and anti-lipid peroxidative effects of the dry matter of the culture broth (DMCB) of *I. obliquus*, Sun et al. (2008) utilized normal, glucose-induced hyperglycemic, and alloxan-induced diabetic mice. The DMCB exhibited a mild hypoglycemic effect in normal mice and achieved euglycemia in glucose-loaded mice after 2 h at a higher dose (1,000 mg/kg compared to 500 mg/kg). In alloxan-induced diabetic mice, the DMCB significantly reduced blood glucose levels, with a notable reduction observed for 21 days. The treatment also decreased serum levels of free fatty acids, total cholesterol, triglycerides, and LDL-cholesterol, while increasing HDL-cholesterol, insulin levels, and hepatic glycogen contents. Additionally, the DMCB enhanced antioxidant enzyme activities and histologically restored pancreas tissues in diabetic mice. Overall, the DMCB of *I. obliquus* demonstrated significant antihyperglycemic, anti-lipid peroxidative, and antioxidant effects in alloxan-induced diabetic mice.

Ma et al. (2013) identified the anti-inflammatory and anticancer compounds present in ethanol, petroleum ether, ethyl acetate, n-butyl alcohol, and water extracts of *I. obliquus*. Among all extracts, the petroleum ether extract was the most active one against human prostatic carcinoma cells and breast carcinoma cell lines with 64.66% and 63.26% inhibitory percentages, respectively. They also isolated lanosterol, 3β-hydroxy-8,24-dien-21-al, ergosterol, inotodiol, ergosterol peroxide, and trametenolic acid from both petroleum ether and ethyl acetate extracts. Among these metabolites, ergosterol, ergosterol peroxide, and trametenolic acid exhibited anti-inflammatory properties, while ergosterol peroxide and trametenolic acid demonstrated cytotoxic effects on human prostatic carcinoma cells and breast carcinoma cell lines. Additionally, these metabolites significantly inhibited nitric oxide production and nuclear factor kappa-light-chain-enhancer of activated B cells (NF-κB) luciferase activity in murine macrophage RAW 264.7 cells.

Xu et al. (2016) discovered flavonoids from ethanol, chloroform, ethyl acetate and n-buthanol extracts of *I. obliquus*. In these extracts epicatechin-3-gallate, epigallocatechin-3-gallate, and naringin, as well as phenolic acids, such as ferulic acid and gallic acid were identified. DPPH radical-scavenging abilities were significantly higher in Tween 20 medium (minimum EC_50_; 40.63 ± 0.89 mg/L) and linoleic acid medium (minimum EC_50_; 43.54 ± 0.92 mg/L) than the control medium (minimum EC_50_; 95.80 ± 1.99).

Baek et al. (2018) isolated triterpenoids from the methanol extract of *I. obliquus* to assess its cytotoxic effects on four human lung adenocarcinoma cell lines, each one with a different p53 tumor protein conditions (human lung adenocarcinoma cell lines A549, H1264, H1299, and Calu-6). They identified several metabolites including 3β-hydroxylanosta-8,24-dien-21, (+)-fuscoporianol C, inonotsutriol E, inotodiol, inonotsutriol A, trametenolic acid, saponaceoic acid I, and a novel lanostane-type triterpenoid, chagabusone A from methanol extracts from *I. obliquus*. Among these, 3β-hydroxylanosta-8,24-dien-21, trametenolic acid, and chagabusone A exhibited the most potent cytotoxicity against all human lung cancer cell lines tested, with EC_50_ values ranging from 75.1 to 227.4 µM. Notably, these compounds reduced the viability of human adenocarcinoma cell lines regardless of p53 mutations or null phenotype. This suggests that the cytotoxic effects observed against human lung cancer cells were independent of p53-related pathways, but rather mediated by apoptosis with caspase-3 activation.

Wold et al. (2020) identified melanin and six triterpenoids from dichloromethane and ethanol extracts of *I. obliquus,* chemically characterized, and evaluated for their anti-inflammatory, immunological, antimicrobial, anticancer, and cytotoxic effects. Among these compounds, melanin, and the triterpenoids 3β-hydroxy-8,24-dien-21-al and inotodiol significantly inhibited the hemolysis of antibody-sensitized sheep red blood cells by human sera. Specifically, 3β-hydroxy-8,24-dien-21-al and inotodiol activated the complement cascade, while the melanin fraction inhibited it. Inotodiol, betulinic acid, and betulin demonstrated anti-proliferative effects against the methotrexate-resistant human adenocarcinoma cell line HT29-MTX (viability: 3.8 ± 0.8 µM, 0.8 ± 0.3 µM, and 1.6 ± 0.4 µM, respectively) and the human lung carcinoma cell line NCI-H460 (viability: 4.7 ± 1 μM, 2.1 ± 0.5 µM, and 2.8 ± 0.4 µM, respectively). Additionally, the melanin fraction and betulinic acid-3-O-caffeate reduced the nitric oxide production in primary murine macrophages. However, these metabolites showed no antimicrobial activity against the tested four bacterial strains (*E. coli*, *S. aureus*, *B. subtilis*, and *P. aeruginosa*) and the yeast *C. albicans*.

Betulin, betulinic acid, inotodiol, and trametenolic acid, extracted with methanol from inner and outer parts (the sclerotium) of *I. obliquus* fruiting bodies, were tested against cancer cell lines (HT-29, AGS, MCF-7, and PC3) by Kim et al. (2020). The MTT assay was conducted to test the effect of triterpenoids on cancer cell lines. They found that the triterpenoids from the outer part extract showed significantly higher anti-proliferative activity against AGS, MCF-7, and PC3 cells compared to the inner part extract.

The hypouricemic properties of triterpenoid acids isolated from *I. obliquus* in mice with hyperuricemia were investigated by Luo et al. (2021). They identified various triterpenoid acids, including 3β,22,24-trihydroxy-lanosterol-8,25-diene, oleanolic acid, 3β-hydroxy-lanoster-8,24-dien-21-acid, 3β,21-dihydroxy-lanosterol-8,24 diene, betulin, inotodiol, 3β-Hydroxy-lanoster-8,24 dien-21-aldehyde, and lanosterol. Their research demonstrated that triterpenoid acids extracted from ethanol extracts of *I. obliquus* effectively inhibited xanthine oxidase activity (with an EC_50_ of 0.065 ± 0.01 mg/mL), displaying a mixed and reversible inhibition pattern. These triterpenoid acids also significantly reduced uric acid levels, hepatic xanthine oxidase, and serum blood urea nitrogen activities in mice with hyperuricemia. This suggests that triterpenoid acids from *I. obliquus* may help suppress kidney damage, lower inflammation in hyperuricemic mice, and exhibit inhibitory effects on xanthine oxidase activity. These findings underscore the potential of triterpenoids derived from *I. obliquus* as a promising dietary or medicinal supplement for managing hyperuricemia.

Zhao et al. (2021) identified flavonoids, including gallic acid, ferulic acid, flavonoids epicatechin-3-gallate, epigallocatechin-3-gallate, naringin, rutin, naringenin, phelligridin G, inoscavin B, and davallialectone, from the ethanol extract of *I. obliquus*. Moreover, they showed that cultivating *I. obliquus* on wheat straw led to increased levels of inoscavin B and davallialectone. In that work, the degradation of lignocellulose boosted the synthesis of flavonoids such as epicatechin-3-gallate, epigallocatechin-3-gallate, rutin, and naringin, thereby enhancing the antioxidative capabilities of *I. obliquus*. The highest antioxidant potential was observed in the extract obtained on day 9 (EC_50_ of 30.96 mg/L) against DPPH radicals.

In a study by Kou et al. (2021), seven newly discovered lanostane-type triterpenoids, named inonotusols H to N, were identified from the ethanol extract of *I. obliquus*. These metabolites exhibited significant inhibition of nitric oxide production in lipopolysaccharide-stimulated BV-2 microglial cells, with EC_50_ values ranging from 2.32 to 23.83 µM. At the concentration of 25.0 µM of these metabolites, no cytotoxicity was observed towards lipopolysaccharide-stimulated BV2 cells. Through molecular docking and Western blotting studies, two of the inonotusols showed the most potent inhibitory effects on iNOS and nitric oxide production. These findings suggest that these bioactive metabolites hold promise for development into therapeutic agents for neurodegenerative disorders, including Alzheimer’s disease.

Abu-Reidah et al. (2021) identified phenolics and flavonoids, including gallic acid, protocatechuic acid, salicylic acid, vanillic acid, 2,3-dihydroxybenzaldehyde, 2,5-dihydroxyterephthalic acid, coumaric acid, caffeic acid, 4-methoxycinnamic acid, hispidin, ferrulic acid, isorhamnetin, myricetin, quercetin, syringic acid, ellagic acid, hispolon, 3,4-dihydroxybenzalacetone, and 3-O-methylellagic acid, from *I. obliquus* extract using the modified Swiss water method. These metabolites showed hydrophilic, lipophilic, and total antioxidant activities.

Li et al. (2021) reported that phelligridin D, extracted from *I. obliquus* using both petroleum ether and ethyl acetate, presented good antioxidant properties. This metabolite reduced reactive oxygen species and malondialdehyde levels while increasing the activity of superoxide dismutase and catalase in human glomerular mesangial cells under high glucose concentration (30 mM). Additionally, it enhanced the capacity of the nuclear factor erythroid 2-related factor 2 (Nrf2), a master transcription factor that upregulates antioxidant response elements (ARE) (Zhao et al., 2017), to promote the transcription of ARE. It was also shown that phelligridin D activated Nrf2 in mesangial cells exposed to high glucose concentration, contributing to its protective effects. Their findings indicated the potential discovery of novel therapies targeting diabetic nephropathy and the applications of *I. obliquus* metabolites in clinical practices.

Wang et al. (2021) identified polyphenol compounds from ethanol extracts of *I. obliquus* such as procyanidin, caffeic acid, p-coumaric acid, isorhamnetin-3-O-glucoside, astilbin, tangeretin, gallic acid, kaempferol, quercetin, and catechin. According to the antioxidant activity of these polyphenols, their DPPH radical scavenging activity increased from 45.12% to 85.64% as the concentration increased from 1.0 to 5.0 mg/mL, respectively. As for their hydroxyl radical scavenging activity, it was found to be 38.76% at 1.0 mg/mL. When the concentration of polyphenols increased from 1.0 to 5.0 mg/mL, its stronger ferric-reducing antioxidant power increased from 0.11 to 0.39 mmol/mL. Their findings suggested that polyphenols from *I. obliquus* possessed promising potential as natural antioxidants.

Chen et al. (2021) extracted forty-six triterpenoids, twelve of which were newly discovered, from *I. obliquus* using ethanol and ethyl acetate stepwise. Among these 46 triterpenoids, thirteen of them showed strong α-glucosidase inhibition, with EC_50_ values from 11.5 to 81.8 µM. This study highlighted the significance of triterpenoids in clarifying the hypoglycemic effects associated with *I. obliquus*.

Peng et al. (2022) showed that ethanol extracts from *I. obliquus*, containing inotodiol, lanosterol, and trametenolic acid, significantly improved lipid accumulation in mouse livers induced by a methionine-choline deficient diet or in human LO2 hepatocyte cells lines induced by oleic acid. These metabolites exhibited protective properties against non-alcoholic fatty liver disease (NAFLD) by mitigating lipid deposition effects, reversing liver weight loss, and reducing liver triglyceride content together with restoring lower levels of alanine transaminase (ALT) and aspartate aminotransferase (AST). Inotodiol specifically demonstrated its anti-NAFLD properties by regulating the lipid metabolism pathway, farnesoid X receptor (FXR)/small heterodimer partner (SHP)/sterol regulatory element-binding protein-1c (SREBP-1c) (Liu et al., 2016). Their findings suggested that these bioactive compounds hold promise as potential drugs for NAFLD treatment.

Ogidi et al. (2018) analyzed raw and fermented ethyl acetate and ethanol extracts of *Lenzites quercina* (*L. quercina*) for their total phenol and flavonoid contents, alongside assessments of their antioxidant properties. The scavenging efficacy of fungal extracts was found against various free radicals, including DPPH, OH^−^, nitric oxide, and Fe^2+^ ranging from 0.12 to 1.80 mg/mL EC_50_ values. Furthermore, petroleum ether, ethyl acetate, and ethanol extracts exhibited EC_50_ lower than the positive controls butylated hydroxytoluene (BHT) and ethylenediaminetetraacetic acid (EDTA). The ethyl acetate extract from fermented *L. quercina* exhibited a higher phenolic content of 67.6 mg GAE/g extract, while the ethyl acetate extract from raw *L. quercina* displayed the highest flavonoid content of 51.4 mg QE/g extract. The antioxidant property, measured by FeCl_3_ reducing power, ranged from 18.1 (fermented *L. quercina* extracted with petroleum ether) to 127.6 mg (raw *L. quercina* extracted with petroleum ether) Ascorbic Acid Equivalent (AAE)/g extract for extracts obtained from both raw and fermented *L. quercina*. Fermented *L. quercina* demonstrated pronounced scavenging properties against nitric oxide and ferrous ion radicals, and it also exhibited superior inhibition of thiobarbituric acid reactive species (TBARS) with the highest inhibitory effect of 109.3%. The study suggested that the high total phenol and flavonoid content in *L. quercina* extracts positioned them as effective antioxidant agents, potentially serving as alternative therapy in healthcare.

A study by Prasher and Manju (2019) analyzed the active constituents present in ethyl acetate, methanol, and hexane extracts of *Peniophora nuda* (*P. nuda*) isolated from mango twigs. GC–MS chromatograms revealed 60, 9, and 60 major peaks in the ethyl acetate, methanol, and hexane crude extracts, respectively. The ethyl acetate extract exhibited 29 peaks with area percentages greater than one, with 13-docosenamide, (Z)- occupying the highest at 12.88%. In the methanolic extract, all 9 peaks had area percentages exceeding 1%, with tricaproin being the highest at 49.82%. The hexane extract displayed 28 peaks with area percentages greater than 1, and 13-docosenamide, (Z)- was the highest at 14.46%. According to their GC-MS findings, *P. nuda* was found to contain significant bioactive compounds with known antioxidant, anti-tumor, antibacterial, immunostimulant, lipoxygenase-inhibitor, anti-aging, analgesic, antidiabetic, anti-inflammatory, antidermatitic, antileukemic, anticancer, hepatoprotective, hypocholesterolemic, antiulcerogenic, vasodilator, antispasmodic, and antibronchitic properties (Prasher and Manju, 2019). These results could serve for identifying and understanding the nature of various bioactive components, with potential applications in biotechnological processes. Further isolation of individual phytochemicals may lead to the discovery of novel drugs. An extensive study of the pharmacological importance, diversity, and chemical composition can provide valuable insights and advance knowledge in this area. Further isolation of bioactive compounds has the potential to reveal new drugs.

The antioxidant properties of terrestrial *Flavodon flavus* (*F. flavus*) and *Xylaria feejeensis* (*X. feejeensis*), harvested from the dry zone forest from Sri Lanka were investigated by Fernando et al. (2016). The study also aimed to determine the contribution of phenolic and flavonoid substances to the antioxidant capabilities of these white rot fungi. Both species exhibited strong antioxidant capacity, indicating the presence of an effective antioxidative system. *F. flavus* demonstrated potent antioxidant activity with an EC_50_ of 77.00 ± 0.18 μg/mL based on DPPH radical scavenging capacity, while *X. feejeensis* exhibited promising antioxidant capacity with an EC_50_ value of 98.4 ± 0.28 μg/mL. Additionally, both analyzed species contained high levels of phenolic and flavonoid substances, suggesting their contribution to the prominent antioxidant activity. *F. flavus* and *X. feejeensis* showed higher total phenol contents of 55.7 ± 10.89 μg gallic acid/mg and 31.33 ± 8.87 μg gallic acid/mg, respectively. They also exhibited elevated levels of total flavonoids, with values of 82.4 ± 4.0 μg epicatechin/mg and 23.35 ± 7.0 μg epicatechin/mg, respectively. Notably, *F. flavus* exhibited a higher amount of total phenolics and flavonoids compared to *X. feejeensis*.

*Fuscoporia torulosa* (*F. torulosa*) is a fungus that develops woody fruiting bodies on both living and deceased trees. From methanol extract of *F. torulosa* fruiting bodies, two distinctive pentacyclic triterpenoids, namely fuscotorunones A and B, were isolated using ethyl acetate and purification by Noji et al. (2021). *In vitro* antimicrobial testing against *B. subtilis*, *S. aureus*, and *C. albicans* was conducted for fuscotorunones A and B. However, the ethyl acetate extract of *F. torulosa* demonstrated antimicrobial activity, with a MIC of 25 μg/mL against *S. aureus* and MIC of 100 μg/mL against *B. subtilis*, fuscotorunones A and B exhibited no activity against all tested microorganisms.

The *Ganoderma* genus belongs to the basidiomycota division, agaricomycetes class, polyporales order and ganodermataceae familiy. Among them, the species *G. lucidum* (Ling-Zhi in Chinese, Reishi in Japanese and Yeongji in Korean) is an outstanding medicinal mushroom having different therapeutical properties. Thus, this fungus is being cultivated worldwide, especially in Southeast Asian countries and many health products are being produced and sold (Bijalwan et al., 2020). Even in Europe there is a biotechnology company named *Hifas da Terra* (https://hifasdaterra.com/en/) that grow some white-rot fungi, *G. lucidum* among them, to extract active biomolecules that are commercialized in different products with diverse benefits for human health (e.g., immune system, oncology, mental health).

Several research works have reported different secondary metabolites from the *Ganoderma* genus, particularly from the *G. lucidum* species (Zhou et al., 2012; Sharma et al., 2019; Lu Y. et al., 2020; Wu et al., 2024), with interesting bioactivities. In this context, Baby et al. (2015) reviewed the biologically active secondary metabolites produced by different species belonging to the *Ganoderma* genus. They stated that phytochemical studies resulted in the isolation of 431 secondary metabolites, from which 240 were isolated from *G. lucidum*. Most of the isolated biologically active secondary metabolites were triterpenes, steroids, and polysaccharides (Seo et al., 2009; Cör et al., 2018). The latter showed to diminish the levels of serum glucose in normal fasted mice after 3 and 6 h of administration (Zhang and Lin, 2004). Likewise, Yang et al. (2007) observed that the administration of a *Ganoderma applanatum* (*G. applanatum*) exopolymer to induced diabetic rats reduced the glucose levels in plasma by 22%. Additionally, it decreased the total levels of cholesterol and triglycerides in plasma by 20.3% and 22.5%, respectively. Also, the activity of alanine transaminase and aspartate transaminase was reduced by 23.2% and 20.7%. Therefore, these compounds could find application to treat diabetes in animals.

Other researchers found that *Ganoderma* extracts presented interesting anti-cancer activities. Thus, for example, lucidenic acids, isolated from triterpenoids of ethanolic extracts of a new *G. lucidum* strain, exhibited anti-invasive activity on human hepatoma carcinoma cells (Weng et al., 2007). Similarly, Li et al. (2013) identified a new triterpenoid, named ethyl lucidenate (ethyl 7β-hydroxy-4,4,14α-trimethyl-3,11,15-trioxo-5α-chol-8-en-24-oate) from ethyl acetate extracts of *G. lucidum* with cytotoxicity against the cancer cell lines HL-60 and CA46. Also, exopolysaccharides obtained from *G. applanatum* presented antitumor activity against carcinoma cell lines (Osińska -Jaroszuk et al., 2014). In addition, *G. lucidum* ganodermic acid was able to inhibit the proliferation of HeLa and U87 human glioma cells, indicating its potential utilization as an anticancer drug (Upadhyay et al., 2014). Li et al. (2018) found that the polysaccharides from *G. lucidum* and *Ganoderma sinense* (*G. sinense*) presented similar chemical characteristics and tumor suppressive activity in mice, which indicated that polysaccharides from *Ganoderma* are therapeutic agents. Also, Wang K. et al. (2019) isolated from ethanolic extracts of *G. lucidum* fruiting bodies 1 new lanostane triterpene and 2 known aromatic meroterpenoids showing high antioxidant and neuroprotective activities. Zhang et al. (2019) showed that ganoderic acids from chloroform extracts of three *Ganoderma* species presented high antiproliferative activity (inhibition percentages from 70.8% to 80.7%) against three cancer cell lines (i.e., gastric carcinoma, liver carcinoma, and colon carcinoma). Bhat et al. (2021) reviewed the bioactivities of polysaccharides produced by different *Ganoderma* genera, mainly by *G. lucidum*, and stated that they presented antitumor, antioxidant, immunomodulatory, antibacterial, neuroprotective, hypoglycemic, and hepatoprotective activities. Therefore, they hold promise for further research to formulate natural efficient drugs to prevent and treat several diseases. More recently, Milhorini et al. (2022) isolated a fucoxylomannan from *G. lucidum* fruting bodies by alkaline extraction with important antimelanomic properties.

On the other hand, there are many research papers reporting antimicrobial activities of *Ganoderma* strains. Thus, Hassan et al. (2019) reported the high antibiotic activity of water extracts from *G. applanatum* against *P. aeruginosa*, *Pseudomonas fluorescens* (*P. fluorescens*), *B. subtilis, Staphylococcus epidermidis* (*S. epidermidis*), and *Micrococcus luteus* (*M. luteus*) strains. In addition, chloroform extracts of *G. lucidum* basidiocarp displayed high antibacterial activity against *Salmonella typhi* (*S. typhi*) (18 ± 2.1 mm DIZ for 100 µL extract) and *B. subtilis* (17 ± 1.9 mm DIZ for 100 µL extract) and high antifungal activity against the yeast *C. albicans* (17 ± 1.7 mm DIZ for 100 µL extract) which was related to their content in polysaccharides and triterpenoids. In addition, *G. lucidum* chloroform extracts also presented high antioxidant activity (Uma Gowrie et al., 2014). Also, methanolic extracts of *G. lucidum* exhibited strong antimicrobial activity against the yeast *S. cerevisiae* (MIC50 value 3 μg/mL) but low antimicrobial activities against Gram-positive bacteria (S*. epidermidis* and *Enterococcus raffinosus* (*E. raffinosus*)) and no activity against Gram-negative bacteria (*E. coli* and *P. aeruginosa*) and the yeast *C. albicans* (Hleba et al., 2014). Also, Ismail et al. (2014) studied the antimicrobial activities of methanol, chloroform, dichloromethane, and hexane extracts of *Ganoderma boninense* (*G. boninense*). The methanol and chloroform extracts showed significant antibacterial activities against different food-borne and skin disease bacterial pathogens (i.e., *E. coli, B. subtilis Bacillus cereus* (*B. cereus*)*, P. aeruginosa, S. pyogenes, Streptococcus pneumoniae, S. aureus, and Klebsiella* spp.). Further, GC-MS results confirmed that *G. boninense* contained bioactive compounds such as dodecanoic acid, cyclododecane, octadecanoic acid, 9-octadecenoic acid, hexadecanoic acid, methyl tetradecanoate, 9, 12-octadecadienoic acid, dodecyl acrylate and hexadecanoic acid. In addition, exopolysaccharides from *G. applanatum* presented antibacterial activity against *S. aureus* (17.98 ± 0.4 mm DIZ and MIC value 1 mg/mL) and toxicity against *V. fischeri* (82.8% cell damage) (Osińska-Jaroszuk et al., 2014). Moreover, ganodermic acid from *G. lucidum* presented antibacterial properties against the Gram-negative bacteria *E. coli* and *P. aeruginosa* (MIC 1 mg/mL) and the Gram-positive bacteria *S. aureus* and *S. epidermidis* (MIC 0.25 mg/mL), pointing out its potential use as a broad-spectrum antibiotic (Upadhyay et al., 2014). Hoque et al. (2015) investigated the antioxidant, antimicrobial and cytotoxic potential of pet ether, chloroform, and methanol extracts of a *G. lucidum* strain collected from Bangladesh. Their results revealed that all the extracts presented high antioxidant activity, low to moderate antibacterial activity (DIZs ranging from 7 mm to 21 mm) against different strains of both Gram-positive (*Sarcina lutea*, *Bacillus megaterium*, *B. subtilis*, *S. aureus,* and *B. cereus*) and Gram-negative bacteria (*P. aeruginosa*, *S. typhi*, *E. coli*, *Vibrio parahemolyticus*, *Vibrio mimicus*, *Shigella boydii,* and *Shigella disenteriae*) and weak cytotoxic activity (brine shrimp nauplii bioassay). However, Romorosa et al. (2017) showed that aqueous extracts of *G. lucidum*, isolated from decaying logs in Isabela State University, Philippines, contained alkaloids, tannins, glycosides and in less extent saponins but not flavonoids. Nevertheless, these extracts had low antimicrobial activity against *E. coli* and especially against *S. aureus*. This was likely because they were devoid in flavonoids. According to the review by Ahmad et al. (2021), *G. lucidum* has a wide range of pharmacological activities, including antiviral activities, due to its content in triterpenoids and polysaccharides. Nonetheless, further studies on the clinical application of the biologically active compounds of this strain are needed. More recently, Chan and Chong (2022) reported the strong antibacterial activity against methicillin-resistant *S. aureus* (MRSA) (DIZ 41.08 ± 0.04 mm and MIC 0.078 mg/mL) of ethyl acetate extracts of a *G. boninense* strain collected from Malaysia. This strong antibacterial activity against MRSA was attributed to its content in aristolochic acid and tamoxifen, which are known to be effective against MRSA (Flores et al., 2016; Bartha et al., 2019), as well as its content in other metabolites with reported antimicrobial properties (i.e., aminoimidazole ribotide, lysine sulfonamide, carbocyclic puromycin, fenbendazole, acetylcaranine, and tigecycline) (Vince et al., 1986; Livermore, 2005; Stranix et al., 2006; Kim et al., 2015; Ločárek et al., 2015; Qadir et al., 2016; Miro-Canturri et al., 2019; de Oliveira et al., 2020). Hence, it could be a promising solution to develop drugs able to fight against multi-antibiotic resistant bacteria. In another recent work, it was shown that hot water extracts of *Ganoderma neo-japonicum* (*G. neo-japonicum*) exhibited 2-fold higher antioxidant and antimicrobial activities (against *S*. *typhimurium*, *Salmonella enteritidis,* and *E. coli*) than those of *G. lucidum* ones. This was presumably related to the higher content in flavonoids of the *G. neo-japonicum* extracts (Ayimbila et al., 2023).

Additionally, Wang et al. (2017) reported anti-aging activities of *G. lucidum* extracts which were mainly exerted through anti-oxidation, immunomodulation, and anti-neurodegeneration. The bioactive compounds responsible for these antiaging effects consisted of polysaccharides, triterpenes, peptides, and polysaccharide peptides. More studies are needed to clarify the mechanisms involved in these antiaging properties.

The *Trametes* genus belongs to the basidiomycota division, agaricomycetes class, polyporales order and family polyporaceae. Different research studies have reported the production of secondary metabolites with various biological activities by several strains of the *Trametes* genus. Among them, *T. versicolor* (also known as *C. versicolor*) is the most studied species. Thus, methanolic extracts of *T. versicolor* exhibited strong antimicrobial activity against the yeast *S. cerevisiae* (MIC50 value 24 μg/mL), low against Gram-positive bacteria (S*. epidermidis* and *E. raffinosus*) and no activity against Gram-negative bacteria (*E. coli* and *P. aeruginosa*) and the yeast *C. albicans* (Hleba et al., 2014). Also, acetonitrile and aqueous extracts of *Trametes hirsuta* (*T. hirsuta*), isolated from decaying logs in Isabela State University, Philippines, presented strong antimicrobial activity against *E. coli* (DIZ 26.36 mm) and *S. aureus* (DIZ 13.87 mm). This could be related to the flavonoid content in the extracts (Romorosa et al., 2017). Furthermore, isolated cerevisterol (ergosta-7, 22E-diene-3β5α, 6β -triol) from methanol extracts of *Trametes gibbosa* (*T. gibbosa*) and *Trametes elegans* (*T. elegans*)*,* collected from farms and forests in Ghana, exhibited a broad-spectrum antibiotic activity. Thus, the isolated cerevisterol from *T. gibbosa* and *T. elegans* inhibited the growth of *S. typhi* (MIC25 and MIC 50, µg/mL, respectively), *S. aureus* (MIC25 and 100 μg/mL, respectively), *A. niger* (MIC25 and 100 μg/mL, respectively) and *E. faecalis* (MIC50 and 200 μg/mL, respectively) (Appiah et al., 2020). Nanglihan et al. (2018) showed that the ethanol extracts of *T. elegans*, collected from the Lingap Kalikasan Park of Central Luzon State University, Philippines, contained flavonoids, tannins, phenols, steroids, alkaloids, anthraquinones, anthrones, coumarins, essential oils, and fatty acids. Also, *T. elegans* extracts presented significant scavenging activity, antibacterial activities against *S. aureus* (DIZ 8.30 mm) and *E. coli* (DIZ 8.07 mm) and high cytotoxicity (brine shrimp nauplii bioassay). Gebreyohannes et al. (2019) found that chloroform, ethanol and hot extracts of two wild fungi, collected from National Reserve Forests, in Kenya, and further identified as *Trametes* spp. showed interesting antimicrobial activities against different test strains (*E. coli*, *K. pneumoniae*, *P. aeruginosa*, *S*. *aureus,* MRSA, *C. albicans,* and *Candida parapsilosis*), the highest one being obtained for *S. aureus* (MIC values 0.83 ± 0.29, 0.67 ± 0.29, and 0.67 ± 0.29 for chloroform, ethanol and hot water extracts, respectively). In addition, Hassan et al. (2019) reported the high antibiotic activity of water extracts from *T. versicolor* against *P. aeruginosa, P. fluorescens, B. subtilis, S. epidermidis, and M. luteus* strains. Bains and Chawla (2020) reported that the methanolic extracts from *T. versicolor*, collected from the forest of Chail in India, contained phenolics as the main compounds followed by flavonoids, ascorbic acid, β-carotene, and lycopene and presented significant antimicrobial activities against *S. aureus, P. aeruginosa*, *K. pneumonia,* and *E. coli* (DIZs ranging from 24.14 to 30.18 mm). It also showed anti-inflammatory activities presumably due to its content in glycopeptides. Furthermore, Oyetayo and Akingbesote (2022) tested the antimicrobial properties of acetone and methanolic extracts from raw and submerged and solid-state fermented *Trametes polyzona* (*T. polyzona*), collected from dead wood in Nigeria, against *S. aureus* isolated from blood, soil, water, and urine. The methanolic extract from submerged fermented *T. polyzona* showed the highest antimicrobial activity against blood isolated *S. aureus* (DIZ 28 mm), probably due to its ability to dissolve the endogenous compounds of the fungus. However, the acetonic extracts presented low antimicrobial activity. GC-MS analysis of *T. polyzona* methanolic extracts showed the following 14 bioactive compounds: caprylic acid methyl ester, tridecanoic acid methyl ester, myristoleic acid methyl ester, cis-10 pentadecanoic acid methyl ester, palmitoleic acid methyl ester, heptadecanoic acid methyl ester, stearic acid methyl ester, elaidic acid methyl ester, oleic acid methyl ester, linolelaidic acid methyl ester, g-linoleic acid methyl ester, x-linolenic acid methyl ester, heneicosanoic acid methyl ester, and cis-11-14-eicosadienoic acid methyl ester. Recently, Begum et al. (2023) reported that the aqueous extracts of *T. hirsuta* exhibited antimicrobial activity against *S. aureus* (DIZ 16.00 ± 0.66 mm for 20 mg/mL extract), *K. pneumonia* (DIZ 14.66 ± 0.88 mm for 20 mg/mL extract) and *Salmonella enterica* (DIZ 13.00 ± 0.88 mm for 20 mg/mL extract). They also related that ethanolic extracts of *T. hirsuta* had significant analgesic, anti-inflammatory and antispasmodic activities. Therefore, *T. hirsuta* could be a valuable source of bioactive compounds to develop new drugs to treat pain, fever and anti-inflammatory disorders, bacterial infections, and gastrointestinal problems. Moreover. Wei et al. (2023) characterized four new sesquiterpenes (three bisabolane sesquiterpenes and one drimane sequisterpene) from *T. versicolor*, one of them (drimarene sequisterpene) showing antimicrobial activity against *S. aureus* (MIC_50_ value 22.2 µM).

On the other hand, Leliebre-Lara et al. (2015) found that n-hexane, dichloromethane, ethyl acetate, and ethanol extracts of *T. versicolor*, collected from a dead and dry trunk in Cuba, presented anti-leishmanial activity against the parasite *Leishmania amazonensis*, being higher in ethyl acetate and ethanol extracts. Also, a partially purified exoproteome of *T. versicolor* culture filtrates highly inhibited the growth and the T2 toxin production of the cereal pathogen *Fusarium langsethiae* (Parroni et al., 2019). Wang K. et al. (2019) reported that the bioactive macromolecule polysaccharopeptide from *T. versicolor* (TPSP), purchased from Fujian Fuzhou Green Valley Biopharmaceutical Technology Research, inhibited the development of morphine addiction in rats. They pointed out that TPSP could be used as an adjunctive therapy approach for the alleviation of morphine resistance in the clinic.

Additionally, a polysaccharide from *Trametes orientalis* (*T. orientalis*) presented chemoprotective effects against cyclophosphamide-induced immunosuppression and oxidative stress in mice (Zheng et al., 2017). Furthermore, Roca-Lema et al. (2019) assessed the anticancer effects of polysaccharide-rich extracts from *T. versicolor* on LoVo and HT-29 human colon cancer cells. Their studies showed that *T. versicolor* extracts inhibited human colon proliferation and cause cytotoxicity. Moreover, blending the extracts with the known anticancer drug 5-fluoruoracil boosted cell cytotoxicity. More recently, He et al. (2021) purified a protein named musarin from *T. versicolor* extract which strongly inhibited the growth of human colorectal cancer cell lines *in vitro*. Therefore, musarin protein holds promise to develop drugs against colorectal cancers, especially against the chemo-resistant ones.

## 3 Concluding remarks

In the evolving landscape of natural bioactive metabolite discovery, white-rot fungi have emerged as prolific sources of novel metabolites, offering versatile applications, including agriculture, healthcare, and pharmaceuticals. These compounds constitute a rich reservoir of bioactive substances, synthesized during secondary metabolism by utilizing intermediate compounds or by-products from primary metabolic pathways. While secondary metabolites are non-essential for an organism’s growth, they show diverse biological characteristics, underscoring their potential significance. White-rot fungi exhibit a unique ability to decompose all wood components, contributing to carbon and nitrogen cycles and producing bioactive substances with several effects, such as antioxidant, antimicrobial, and anticancer properties. In light of explanations, this article reviews the potential application of biologically active secondary metabolites from white-rot fungi in different fields like nutrition, medicine, and degradation. As shown, the diversity of these compounds highlights their importance in forthcoming research, advancements, and practical applications across various industries. This underscores the crucial contribution that white-rot fungi can make in influencing the field of biotechnology and sustainable development. Nevertheless, scaling up production on a large scale is necessary to assess the feasibility of commercial applications.
